# Defining the role of corticotropin releasing factor binding protein in alcohol consumption

**DOI:** 10.1038/tp.2016.208

**Published:** 2016-11-15

**Authors:** C L Haass-Koffler, A T Henry, G Melkus, J A Simms, M Naemmuddin, C K Nielsen, A W Lasek, M Magill, M L Schwandt, R Momenan, C A Hodgkinson, S E Bartlett, R M Swift, A Bonci, L Leggio

**Affiliations:** 1 Section on Clinical Psychoneuroendocrinology and Neuropsychopharmacology, NIAAA DICBR and NIDA IRP, NIH, Bethesda, MD, USA; 2 Center for Alcohol and Addiction Studies, Department of Psychiatry and Human Behavior, Brown University, Providence, RI, USA; 3 Center for Alcohol and Addiction Studies, Department of Behavioral and Social Sciences, Brown University, Providence, RI, USA; 4 Department of Psychology, University of Chicago, Chicago, IL, USA; 5 Department of Radiology, University of Ottawa, Ottawa, ON, Canada; 6 Department of Neurology, University of California, San Francisco, San Francisco, CA, USA; 7 Department of Psychiatry, University of Illinois, Chicago, IL, USA; 8 Office of the Clinical Director, NIAAA, NIH, Bethesda, MD, USA; 9 Clinical Neuroimaging Research Core, NIAAA, NIH, Bethesda, MD, USA; 10 Laboratory of Neurogenetics, NIAAA, NIH, Rockville, MD, USA; 11 Queensland University of Technology, Brisbane, QLD, Australia; 12 NIDA Intramural Research Program and Departments of Neuroscience and Psychiatry, Johns Hopkins University, Baltimore, MD, USA

## Abstract

The corticotropin releasing factor (CRF) exerts its effects by acting on its receptors and on the binding protein (CRFBP), and has been implicated in alcohol use disorder (AUD). Therefore, identification of the exact contribution of each protein that mediates CRF effects is necessary to design effective therapeutic strategies for AUD. A series of *in vitro/in vivo* experiments across different species were performed to define the biological discrete role of CRFBP in AUD. First, to establish the CRFBP role in receptor signaling, we developed a novel chimeric cell-based assay and showed that CFRBP full length can stably be expressed on the plasma membrane. We discovered that only CRFBP(10 kD) fragment is able to potentiate CRF-intracellular Ca^2+^ release. We provide evidence that *CRHBP* gene loss increased ethanol consumption in mice. Then, we demonstrate that selective reduction of *CRHBP* expression in the center nucleus of the amygdala (CeA) decreases ethanol consumption in ethanol-dependent rats. CRFBP amygdalar downregulation, however, does not attenuate yohimbine-induced ethanol self-administration. This effect was associated with decreased hemodynamic brain activity in the CRFBP-downregulated CeA and increased hemodynamic activity in the caudate putamen during yohimbine administration. Finally, in alcohol-dependent patients, genetic variants related to the CRFBP(10 kD) fragment were associated with greater risk for alcoholism and anxiety, while other genetic variants were associated with reduced risk for anxiety. Taken together, our data provide evidence that CRFBP may possess both inhibitory and excitatory roles and may represent a novel pharmacological target for the treatment of AUD.

## Introduction

Stress plays an important role in the development and maintenance of alcohol use disorder (AUD).^1^ In response to stress, corticotropin releasing factor (CRF) activates the hypothalamic−pituitary−adrenal axis (HPA), leading to the subsequent release of glucocorticoids.^2^ Additionally, CRF mediates behavioral responses to stress via extrahypothalamic regions. CRF exerts its effects via binding to its receptors (CRFR1 and CRFR2), and to the 37 kD CRF binding protein (CRFBP).^3^ Compared to CRF and its receptors, CRFBP has been much less investigated. This is due to the lack of control of spontaneous CRFBP proteolytic cleavage that yields a N-terminal fragment (27 kD) and a C-terminal fragment (10 kD), and the difficulty of purifying large-enough quantities of CRFBP 37 kD full length (FL) for *in vitro* and *in vivo* experiments.^4^

Early preclinical studies showed that CRFBP is expressed in the cerebral cortex, subcortical limbic structures and anterior pituitary corticotropes, suggesting that CRFBP plays a critical role in modulating endocrine and behavioral responses to stress.^5^ Recent evidence shows that CRFBP and CRFR2α co-exist in rat ventral tegmental area (VTA) glutamatergic/GABA synaptosomes that originate from hypothalamic areas.^6^ Using microinjection of the CRFBP antagonist CRF_6−33_ in the VTA, it was also demonstrated that CRFBP, via CRFR2, has a pivotal role in escalation of ethanol drinking.^7^ In line with this recent study, earlier work demonstrated that CRF-mediated cocaine positive reinforcement is associated with CRFBP and CRFR2 interaction in the VTA dopamine system.^8^ All these data support our hypothesis that CRFBP is not only a sequestering protein, but it may possess additional functions. Our previous electrophysiology work demonstrated that in the VTA CRF modulates neuronal excitability, through CRFBP and CRFR2 interactions, by potentiating *N*-methyl-d-aspartate-mediated excitatory postsynaptic currents.^9^ Therefore, elucidation of the selective molecular functional of CRFBP and its interaction with CRFR2 may help us understand the mechanisms underlying the role of CRFBP in alcohol consumption, thus facilitating the potential development of effective treatments targeting the CRFBP in AUD.

## Materials and methods

### Subjects

All animal procedures were approved by the Ernest Gallo and Clinical Center Institutional Animal Care and Use Committee, were in accordance with NIH guidelines for the Humane Care and Use of Laboratory Animals, and were conducted in agreement with the Guide for the Care and Use of Laboratory Animals, National Research Council, 1996. Human subject data were collected according to screening and assessment NIAAA protocols approved by the appropriate NIH institutional review boards. All participants provided written consent before participating. Subjects' description is detailed in the Supplementary information.

### Procedures

#### Transfection and expression of human cells with the CRFBP-CRFRs chimera

The plasmids containing the CRFBP-CRFRs, detailed in Supplementary information, were transfected into HEK293 cells to verify expression of the protein, confirm appropriate insertion into the membrane, and determine the functionality of the chimera. Prior to transfection, human cells were placed in a six-well plate, with DMEM and 10% fetal bovine serum in each of the wells, and checked for 90–95% cell confluency. The media was aspirated from the wells and replaced with fresh DMEM/10% fetal bovine serum. The final DNA plasmid preparation was then diluted in Opti-MEM reduced serum. Lipofectamine 2000 was mixed in Opti-MEM and incubated at room temperature for 5 min, and then combined with the diluted DNA. Cell selection, flow cytometry, immunofluorescence, image analysis, quantification of receptors' surface expression and fluorescence-based Ca^2+^ assay are described in the Supplementary information.

#### Lentivirus expressing shRNA targeting *CRHBP*

We designed three sets of shRNAs corresponding to different regions on the *CRHBP* mRNA sequence and cloned DNA oligonucleotides encoding the shRNAs into the lentiviral vector pLL3.7,^10, 11^ which also expresses GFP. pLL3.7 is available from the plasmid repository Addgene (Cambridge, MA, USA, plasmid #11795). The shRNA sequences are designated by the number of the first base of the 19-nucleotide targeting sequence in rat *CRHBP* (NCBI NM_139183.2): 1050 (5′-GAAACAGCATCCCGGAGTA-3′), 937 (5′-GAAAATTAGCTGCGACAAT-3′) and 240 (5′-GCGCCAATTTGAAGCGGAA-3′). As a control, we used a sequence that did not recognize any known mammalian gene in a BLAST search (Scr, 5′-GCGCTTAGCTGTAGGATTC-3′). Lentivirus was produced using the ViraPower Lentiviral Packaging Mix (Thermo Fisher Scientific, Waltham, MA, USA). Viral titers were determined by p24 enzyme-linked immunosorbent assay (Zeptometrix, Buffalo, NY, USA) and were ~8 × 10^7^ pg ml^−1^ for viruses encoding control and *CRHBP* shRNAs. The knockdown efficiency, intra-amygdala infusion of lentivirus, rats' self-administration apparatus and training are described in the Supplementary information.

#### Drinking in the dark procedure

Male, mixed-genetic-background (C57BL/6/SV129 mix) mice were all weaned at P21 and entered in the drinking-in-the-dark (DID) cycle protocol at greater than 6 weeks of age. After a 2-week acclimation period, *CRHBP*−/− and *CRHBP*+/+ littermates were given access to one bottle of 20% (v/v) ethanol for 2 h, for three consecutive days; access began 3 h into the dark cycle. On the fourth consecutive day, mice were given access to one bottle of 20% (v/v) ethanol for 4 h. The procedure was repeated each week, with 3 days between cycles. This DID procedure was used due to the high intake of ethanol on the fourth day of access.^12^

#### fMRI protocol

To determine if local knockdown of amygdala *CRHBP* by RNA interference produces functional differences compared to control rats, we measured the hemodynamic activity related to neural activity by blood-oxygen-level dependence after pharmacological stimuli (yohimbine) using functional magnetic resonance imaging (fMRI). In brief, during the magnetic resonance imaging (MRI) scan, after intraperitoneal (i.p.) saline infusion, all animals received yohimbine (i.p. 2 mg kg^−1^, vol. 5 ml kg^−1^). Data acquisition and post-processing are described in the Supplementary information.

### Data analysis

Distributional characteristics of outcome measures were examined to evaluate similarity to the normal distribution. The Generalized Estimating Equation (GEE) model was utilized to analyze the drinking behavior of all animals and to compare the hemodynamic activity between *CRHBP* shRNA rats and *Scr* shRNA rats. GEE was chosen because it can handle unmeasured dependence when outcomes are repeated measures. We selected GEE over repeated-measures analysis of variance because it can more flexibly handle a number of data needs (for example*,* missing data, assumed auto correlation structure).

In the GEE model, for the *CRHBP*-deficient mice analysis was included the main effect of genotype (*CRHBP*−/− mice compared to *CRHBP*+/+ littermates), main effect for time, defined as the six ethanol exposures in the DID paradigm, and finally genotype × time interaction. For the *CRHBP* knockdown rats in the ethanol self-administration procedure, the GEE model included the main effect of CRFBP downregulation by shRNA (*CRHBP* shRNA compared to *Scr* shRNA), main effect of time, defined as the 25 ethanol exposures in the operant chamber after surgery, and finally shRNA × time interaction. For the fMRI procedure using *CRHBP* knockdown rats, the GEE model included the main effect of shRNA, main effect of yohimbine (compared to saline), main effect of time, defined as the time during the fMRI procedure comprised by saline (20.8-41.5 min) and yohimbine (41.6-62.4 min) infusion corrected from baseline (0-20.7 min), and shRNA × yohimbine interaction. The same model was used to analyze center nucleus of the amygdala (CeA), paraventricular nucleus (PVN) and caudate putamen. *Post hoc* analysis was used to assess specific time-point differences over time and *P*-values were *Bonferroni* corrected for multiple comparisons. In *CRHBP*−*/*− mice and their *CRHBP+/*+ littermates, unpaired *t*-test was utilized to measure blood ethanol content level, water or sucrose consumption on day 4, the amount of *pro*-CRF to detect the differences among genotypes in the PVN and CeA. In the *CRHBP* shRNA analysis, unpaired *t*-test was utilized to analyze the *in vitro* downregulation, rats' ethanol self-administration after recovery from surgery, and hemodynamic activation between saline and yohimbine infusion. Also, for Ca^2+^ assay signaling, comparisons between groups were performed using unpaired *t*-test. *Pearson* correlation was utilized to evaluate ethanol consumption and blood ethanol content.

Allele frequencies and genetic association analyses in humans were conducted using PLINK v1.07.^13^ Association with alcohol dependence diagnosis was tested using logistic regression and an additive genetic model. Further tests were conducted to evaluate associations with alcohol drinking and anxiety phenotypes in alcohol dependent individuals using linear regression. All models were conducted in the full sample, as well as split by European and African ancestry based on subject self-report. Age, gender, and ancestry informative marker scores for European and African ancestry were included as covariates in all models, even when split by ancestry; total score from the Childhood Trauma Questionnaire was also included as a covariate in the linear regression models for drinking and anxiety outcomes.

Results are reported as mean (*M*) and error bars indicate standard error (s.e.m.). All statistical tests were two-sided, and statistical significance was accepted if a *P-value < 0.05 was obtained. SPSS (v.22) (Armonk, NY, USA) was used to conduct the analysis and GraphPad Prism (v.5) was used to generate figures (La Jolla, CA, USA).

## Results

### CRFBP(FL) and its fragments, CRFBP(27kD) and CRFBP(10 kD), are stably expressed on the plasma membrane of both CRFR2α and CRFR1

**Figure 1:**
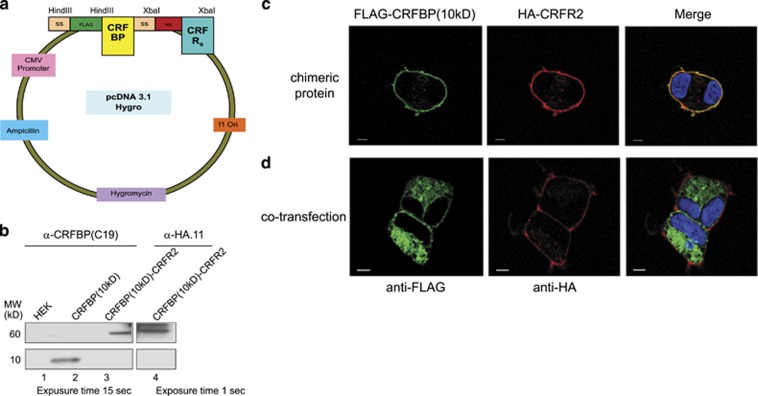
The CRFBP(10 kD)-CRFR2α chimeras are expressed on the plasma membrane of HEK293 cells. (**a**) Schematic representation of the FLAG (DYKDDDDK epitope)-tagged CRFBP with hemagglutinin (HA)-tagged corticotropin releasing factor receptors (CRFRs) cloned into pCDNA3.1-based mammalian expression vector. (**b**) Western blot analysis of FLAG-CRFBP(10 kD)-HA-CRFR2α chimeric constructs. The blots were probed with primary antibody: anti-CRFBP C-19 (1:100) or anti-HA.11 (1:1000), then probed with secondary antibody: donkey anti-goat (IgG-HRP) (1:5000) to visualize CRFBP, or goat anti-mouse (IgG H+L-HRP) (1:5000) to visualize the receptors. Lane 1: HEK293 cells (negative control), lane 2: FLAG-CRFBP(10 kD) (MW~10 kD) (positive control), lane 3: FLAG-CRFBP(10 kD)-HA-CRFR2α (MW~60 kD), lane 4: FLAG-CRFBP(10 kD)-HA-CRFR2α (MW~60 kD). Immunohistochemical staining of HEK293 cells: (**c**) transfected with FLAG-CRFBP(10 kD)-HACRFR2α showing CRFBP(10 kD) stably expressed on the cell membrane and co-expressed with CRFR2α. The cells were fixed and permeabilized and then probed with anti-FLAG antibodies for FLAG-CRFBP(10 kD) and visualized using AlexaFluor-488 conjugated anti-mouse (IgG_2a_) secondary antibody, or probed using anti-HA and visualized using AlexaFluor-594 conjugated anti-mouse (IgG_1_) antibody and merged fluorescent image with 4',6-diamidino-2-phenylindonle (DAPI) to visualize the nuclei; (**d**) co-transfected with CRFBP(10 kD) with HA-CRFR2α, which does not interact with the receptor at the plasma membrane. FLAG-CRFBP(10 kD) visualized using AlexaFluor-488 conjugated anti-mouse (IgG_2a_) secondary antibody was co-transfected into HEK293 cells with HA-CRFR2α, probed using anti-HA, and visualized using AlexaFluor-594 conjugated anti-mouse (IgG_1_) and merged fluorescent image with DAPI to visualize the nuclei. Scale bar, 5 μM.

Purifying human CRFBP or its fragments in sufficient quantities for investigation has not been successful to date. The precise mechanism that promotes CRFBP(FL) cleavage into two fragments, CRFBP(27kD) and CRFBP(10 kD), is still under investigation.^4^ Our goal was to have CRFBP(FL) and its fragments stably expressed and maintained in close proximity to the receptors in order to determine whether they modulate the receptors' signaling. To achieve this expression, we created six covalently linked polypeptides between CRFBP, and its fragments, to the receptors. We fused FLAG-tagged CRFBP(FL), CRFBP(27kD) or CRFBP(10 kD) with HA-tagged CRFR1 or HA-CRFR2α and cloned these into a pCDNA3.1-based mammalian expression vector (Figure 1a). Constructs were verified by sequencing, protein integrity was verified by western blot (Figure 1b, Supplementary Figure S1a) and protein expression was confirmed using immunofluorescence: FLAG-CRFBP(10 kD)-HA-CRFR2α (Figure 1c, Supplementary Movie S1), FLAG-CRFBP(FL)-HA-CRFR1, FLAG-CRFBP(FL)-HA-CRFR2α, FLAG-CRFBP(27kD)-HA-CRFR1, FLAG-CRFBP(27kD)-HA-CRFR2α and FLAG-CRFBP(10 kD)-HA-CRFR1 (Supplementary Figure S1b). We also co-transfected FLAG-CRFBP(10 kD) (Figure 1d), FLAG-CRFBP(FL) and FLAG-CRFBP(27 kD) (Supplementary Figure S1c) with HA-CRFRs into HEK293 cells as an additional control, to verify that CRFBP and its fragments, when not tethered to either CRFRs, were expressed intracellularly in regulated secretory pathways and did not interact with the receptors on the cell membrane.

### Only CRFBP(10 kD)-CRFR2α can potentiate CRF-induced CRFR2α signaling

**Figure 2:**
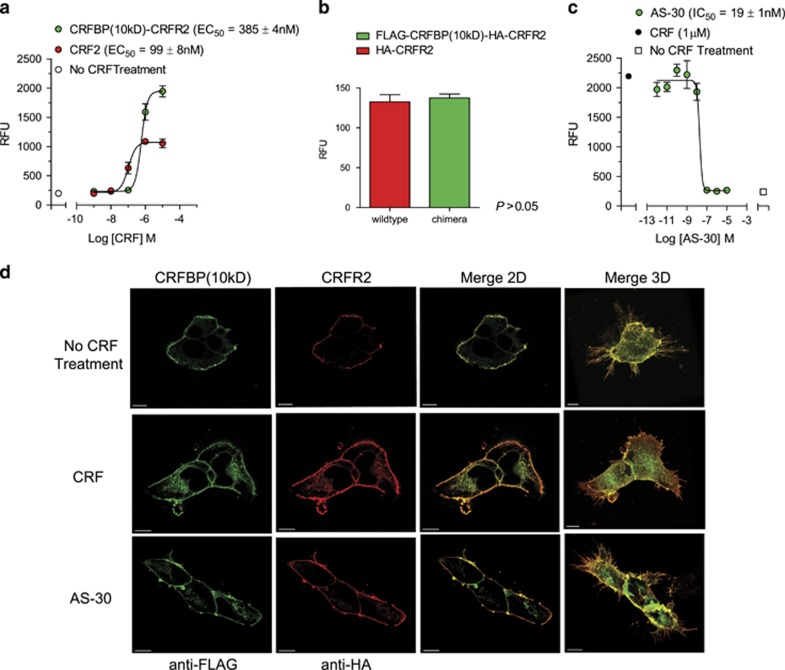
The CRFBP(10 kD)-CRFR2α chimeras potentiate CRF-induced release of intracellular Ca^2+^. (**a**) Full-dose response curves were generated for CRF-induced release of intracellular Ca^2+^ at the CRFBP(10 kD)-CRFR2α chimera (EC_50_=385± 4.0  nM) compared with CRFR2α-only transfected cells (EC_50_=99±8 nM). From comparison of the Emax values, we determined that CRFBP(10 kD), when tethered to CRFR2α (*E*_max_=2520±85 RFU), potentiates CRF-induced signaling when compared to the CRFR2α-only signal (*E*_max_=930±81 RFU, ***P>0.05). (**c**) The CRFR2 antagonist, antisauvagine-30 (AS-30, 1 pm−10 μM), inhibits CRF (1 μm)-induced intracellular calcium release in HEK293 cells stably expressing FLAG-CRFBP(10 kD)-HA-CRFR2α (IC_50_=5 3±0.4 nM). (**d**) Immunocytochemical analysis of receptor trafficking. Cells were left untreated (NT); treated with the agonist (CRF) for 30 min (1 μM, promoted endocytosis of CRFR2α and the extracellular receptors were contracted); pretreated with CRFR2 selective antagonist AS-30 for 30 min (1 μm) and then treated with agonist (CRF). Antagonist with AS-30 reduced CRF-induced endocytosis; cells showed no significant difference in receptor contraction compared to cells left untreated. Scale bar, 5 μM. Results are expressed as *M*±s.e.m., relative fluorescence units (RFU), calculated as agonist-induced maximum calcium peak/cell number × 1000. CRFR, corticotropin releasing factor receptor.

Next, we examined the functional properties of the covalently linked polypeptides created from CRFBP. We tested the ability of the chimeric proteins to activate intracellular calcium (Ca^2+^) release to evaluate if they are capable of producing CRF-induced receptor signaling. Full-dose response curves were generated for CRF-induced release of intracellular Ca^2+^ at all chimera proteins and compared with HA-CRFR-only transfected cells. From comparison of the E_max_ values, we determined that only FLAG-CRFBP(10 kD) when tethered to HA-CRFR2α potentiates CRF-induced signaling, when compared to the CRFR2α-only signal (Figure 2a). The activation by CRFBP(10 kD) via CRFR2α, which leads to increased signaling in response to CRF, was unique to the combined action of CRFR2α tethered to the CRFBP(10 kD) fragment, was not mimicked by the other chimeric proteins (Supplementary Figures S2a–c), and it was not due to the different levels of functional protein delivered to the cell surface (Figure 2b). In particular, CRF binds both CRFR1 and CRFR2α, but is more potent at CRFR1.^14, 15, 16, 17^ Interestingly, there is ample evidence that CRFR1 is associated with anxiety-related behaviors.^18, 19^ In light of this, we examined whether the observed enhancement of the CRFBP(10 kD) chimeric complex signaling was specific to CRFR2α or whether the expression of CRFR1 would also increase signaling in a chimeric protein when tethered with CRFBP(10 kD). As expected, CRF induced a potent release of intracellular Ca^2+^ at CRFR1; however, there was no enhancement of CRF-induced signaling at both the CRFBP(10 kD)-CRFR1 and CRFBP(27kD)-CRFR1 chimeric proteins (Supplementary Figure S2b).

In additional control experiments, CRF-mediated Ca^2+^ signaling was absent in any FLAG-CRFBP-only transfected cells, and in non-transfected HEK293 cells, which do not express endogenous CRFR1 or CRFR2α (Supplementary Figures S2d–h). To further test the full functionality of the FLAG-CRFBP(10 kD)-HA-CRFR2α chimeric protein, we examined whether it can be also inhibited by the CRFR2 antagonist antisauvagine (AS-30). The Ca^2+^ assay experiment demonstrated that the increase in intracellular Ca^2+^ release elicited by 1 μM CRF (EC_80_) was dose-dependently inhibited by AS-30 (IC_50_) (Figure 2c).

Finally, the complete functionality of FLAG-CRFBP(10 kD)-HA-CRFR2α chimera was tested by evaluating its post-endocytic behavior. A three-dimensional (3D) confocal analysis showed chimeric receptors were distributed in the plasma membrane and projected outward from finite regions of the membrane of the transfected cells (Figure 2d). When cells transfected with FLAG-CRFBP(10 kD)-HA-CRFR2α were left untreated, there was no significant receptor internalization. CRF-induced internalization of FLAG-CRFBP(10 kD)-HA-CRFR2α was inhibited by pretreatment with the CRFR2 selective antagonist AS-30.

### Higher ethanol consumption in *CRHBP*−*/*− mice compared to their *CRHBP+/+* littermates

**Figure 3:**
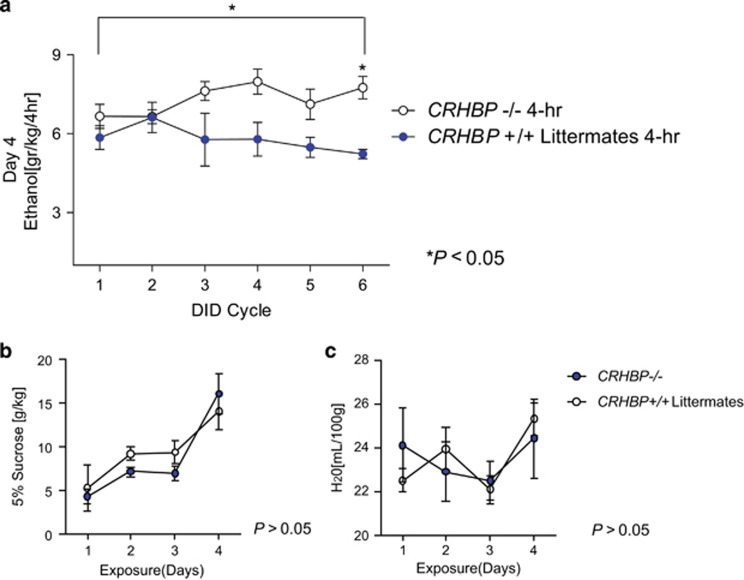
Ethanol consumption is significantly higher in *CRHBP−/−* mice compared to their *CRHBP+/+* littermates in the drinking in the dark (DID) procedure. (**a**) During the 4-h period on day 4, there was a significant main effect of genotype (*CRHBP−/− vs CRHBP+/+*; *n*= 17−16, respectively; *B*= 0.977, *P< 0.05), no main effect of time (*n*= 17−16, *P*>0.05), but a trend of genotype × time interaction (*n* = 17−16, B_1_= 0.289, P = 0.073) with ethanol consumption increased after repeated DID exposures and a strong effect at the sixth DID cycle. There was no difference in (**b**) 5% (v/v) sucrose consumption (*CRHBP−/− vs CRHBP+/+*; *n*= 10−4, *P*>0.05) and (**c**) total water consumption (*CRHBP−/− vs CRHBP+/+*; *n*= 8−3, *P*>0.05), between the *CRHBP−/−* mice and their *CRHBP*+/+ littermates. Results are reported as *M*±s.e.m.; not significant (*P*> 0.05).

To investigate the *in vivo* role of CRFBP in binge-like alcohol consumption, we tested the well-validated *CRHBP*-deficient (−/−) mouse model ^20^ with the DID procedure using a 6-week paradigm.^12^ Wild-type (*CRHBP+/+*) littermates, used as control, underwent the same procedures. There was a significant main effect of genotype in increasing ethanol consumption in the *CRHBP*−/− mice, compared to *CRHBP*+/+ littermates during the 4-h period on day-4 of ethanol access (Figure 3a, Supplementary Table S1). There were no main effects of time; however, we found a trend of genotype × time interaction. *Post hoc* analysis revealed that ethanol consumption increased after repeated DID exposure, with a strong effect at the 6-DID cycle.

There was no difference between genotypes in 5% (v/v) sucrose consumption in the DID model (Figure 3b), nor in total water consumption during the 21-hour period when mice were not exposed to ethanol (Figure 3c). Furthermore, consistent with previous DID studies,^12^ there was a linear correlation between ethanol intake and blood ethanol content in both genotypes. At baseline, the two genotypes consumed similar amounts of ethanol and we found no difference in blood ethanol content between *CRHBP*−/− and *CRHBP+/+* littermates (Supplementary Figures S3a and b). To evaluate if there were CRF compensatory effects in the *CRHBP−/−* mice, we measured the amount of CRF from brain homogenates in the PVN of the hypothalamus and in the CeA. Western blot of *pro*-CRF expression in the PVN, where CRF is synthesized,^21^ and in the CeA, where it is highly expressed,^22^ at week 1 of the DID procedure in ethanol naive mice showed no significant difference between the genotypes (Supplementary Figure S3c). Taken together, these results indicate that there were no baseline compensatory effects in *CRHBP−/−* mice that might account for the difference in ethanol consumption compared to wild-type littermates.

### CRFBP downregulation in the CeA reduces ethanol self-administration and CeA hemodynamic activity, but does not attenuate yohimbine-induced ethanol self-administration

Previous studies have reported human (h)CRFBP mRNA and protein level in rat brain^5^ and dense hCRFBP-stained terminal fields in the CeA of human brain.^23^ However, very little is known about the mechanism and molecular network that underpin the stress-related functions mediated by CRFBP in the CeA, a limbic region that controls emotionality and alcohol reinstatement.^24, 25^ Therefore, to investigate the causal link between CRFBP in the CeA and alcohol-seeking behaviors, we utilized a rat model trained to self-administer ethanol, designed with controlled regional expression of CRFBP. Sequences encoding small hairpin RNAs (shRNAs) targeting *CRHBP* were cloned into a lentiviral vector. We tested for the ability of the shRNAs to knock down *CRHBP* expression *in vitro*. We found that CRFBP expression was significantly reduced in cells infected with lentivirus expressing *CRHBP* shRNA, as compared to cells infected with the control non-targeting shRNA (*Scr)* (Supplementary Figures S4a-c). We then selected the most potent *CRHBP* shRNA for *in vivo* testing. Long-Evans rats trained to self-administer ethanol were injected bilaterally in the CeA with lentivirus expressing the *CRHBP* or the control *Scr* shRNA. We confirmed infection in the CeA *in vivo* by magnetic resonance imaging scan (Supplementary Figure S4d) and *post-mortem* by GFP visualization of infected cells in each animal.

After recovery from surgery, during the subsequent 15 ethanol sessions, a significant shRNA × time interaction was found. *Post hoc* analysis indicated that rats infected with *CRHBP* shRNA showed a significant reduction in ethanol consumption. By contrast, in the *Scr* shRNA control rats, there was no statistical difference between the amount of ethanol consumed at baseline and the following sessions (Supplementary Figure S4E). Reduction of CRFBP expression in the CeA did not however attenuate yohimbine-induced ethanol drinking, as both *CRHBP* shRNA and *Scr* shRNA rats increased ethanol consumption after yohimbine i.p. administration. Taken together, these results indicate that *CRHBP* shRNA rats were unable to return to their baseline self-administration drinking behaviors. However, this effect disappeared after administering a pharmacological stressor via a yohimbine challenge.

**Figure 4:**
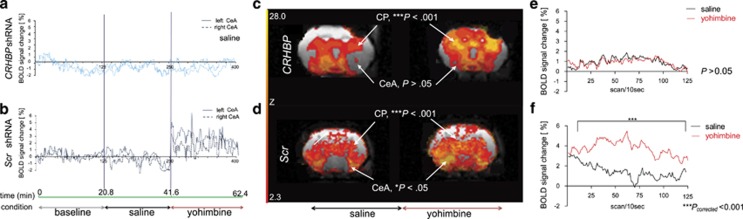
Brain hemodynamic activity in the center nucleus of the amygdala (CeA) downregulated CRFBP in rats trained to self-administer ethanol. *CRHBP* short hairpin RNA (shRNA) (upper panel) and *Scr* shRNA (lower panel). (**a, b**) CeA bilateral representative functional magnetic resonance imaging scans of *Scr* shRNA controls and *CRHBP* shRNA rats. Timeline (green line) and pharmacological experimental manipulation (black: baseline; blue: saline; red: yohimbine). (**c, d**) We found a strong main effect of yohimbine in the caudate putamen (*n*=6 each group*, B*_1_=−0.801, ***P<0.001) and a trend of main effect of yohimbine in the PVN (*n*= 6 each group*, B*_1_= 0.8876, *P*= 0.069). There was no main shRNA effect or yohimbine × shRNA interaction for these outcomes. In the CeA, when we compared the shRNA rats and condition (saline and yohimbine) effects, we found a main significant effect of yohimbine (*n*=6 each group*, B*_1_=1.125, *P< 0.05) and a shRNA × yohimbine interaction (*n*= 6 each group, *B*_1_= −2.372, *P< 0.05). A Bonferroni *post ho*c analysis revealed that (**e**) in the *CRHBP* shRNA rats there was no difference in CeA activation between saline and yohimbine infusion (*P*_corrected_*>*0.05), but (**f**) there was a stronger activation in the control *Scr* shRNA rats (*t*_1499_= 2.511, ***P_corrected_< 0.001). Coronal sections are presented from their antero-posterior orientation. Results are reported as *M*±s.e.m..; not significant (*P*> 0.05). PVN, paraventricular nucleus.

To further validate the *in vivo* downregulation of CRFBP and the effect of yohimbine administration, we evaluated intrinsic brain excitability using blood-oxygen-level dependence, a measure of neural hemodynamic activity. During the magnetic resonance imaging scan, after a baseline assessment, all animals received a saline, followed by a yohimbine, i.p. infusion^25^ (Figure 4a and b). To make a mechanistic link between the effect of CRFBP knockdown in the CeA and ethanol self-administration, we analyzed other brain regions that are known to show activation during yohimbine administration. Yohimbine produced a composite pattern of blood-oxygen-level dependence % signal change between the *CRHBP* and the *Scr* shRNA rats in the limbic, hypothalamic and subcortical areas (Figure 4c and d). We found a main effect of yohimbine in the caudate putamen and a trend in the PVN. There was no main shRNA effect or yohimbine × shRNA interaction in these brain regions. In the CeA, there was a lower hemodynamic activation in *CRHBP* shRNA rats compared to controls. Furthermore, when we compared the shRNA rats (*CRHBP* vs *Scr*) and condition (saline vs yohimbine) effects, we found a main significant effect of yohimbine and a shRNA × yohimbine interaction. *Post ho*c analyses revealed that there was no difference in CeA activation between saline and yohimbine infusion in the *CRHBP* shRNA rats (Figure 4e). By contrast, as expected, there was a significant difference in CeA activation between saline and yohimbine infusion in the control *Scr* shRNA rats (Figure 4f).

### Human genetic association study of CRFBP(10 kD) candidate gene variants in alcoholic individuals

Consistent with previous rodent work, the *in vivo* experiments above indicate a role of CRFBP in alcohol-seeking behaviors. Furthermore, the *in vitro* chimeric work demonstrates a peculiar role of the CRFBP(10 kD) fragment in potentiating CRF signaling. Finally, a human genetic study was conducted in order to provide preliminary translation of these *in vivo* and *in vitro* findings.

We *a priori* selected single-nucleotide polymorphisms (SNPs) of the *CRHBP* gene that are located in gene regions related to CRFBP(10 kD), an approach driven by the chimeric results reported above and were investigated for possible association with alcoholism in previous studies,^26, 27^ an approach driven by the rodent results reported above. This approach led to the selection of three SNPs located in the CRFBP(10 kD) gene regions: *rs10055255* and *rs10062367,* which were previously evaluated in stress-induced alcohol craving in heavy drinkers;^26^ and *rs7728378,* which was previously investigated in Caucasian alcohol dependent individuals.^27^ Notably, the latter SNP was associated with suicide attempt in another study of patients with alcohol and substance dependence.^28^ Only *rs10062367* was present in our dataset. For the other two SNPs, we were able to identify proxies (*rs1053989* and *rs7718461*) in high linkage disequilibrium, in both the European and African subsamples (an important aspect given that the dataset includes approximately 60% Caucasians and 40% African Americans, Supplementary Table S2).

While there were no differences in the prevalence of these three SNPs analyzed between alcohol dependent patients and controls (data not shown), the *rs10062367* A allele showed a significant association with average drinks per drinking day, both in the full sample as well as in the African ancestry subgroup. Associations between the three SNPs and alcohol phenotypes in subjects with current alcohol dependence are summarized in Table 1 and allele frequencies in Supplementary Table S3. For the anxiety measures, there was a significant main effect of *rs10062367* A allele for the State-Trait Anxiety Inventory anxiety in European ancestry. We found a significant effect of *rs7718461* A allele for the Comprehensive Psychopathological Rating Scale anxiety in European ancestry, and a negative association with this scale anxiety in European ancestry. There was a significant effect of *rs1053989* C allele for the Comprehensive Psychopathological Rating Scale anxiety in the European ancestry. Finally, we found a significant effect for neuroticism in the European ancestry. These results suggest the possibility that variation of the CRFBP(10 kD) protein might alter the risk of alcohol drinking and/or anxiety in alcohol-dependent patients.

## Discussion

Using a combination of different *in vitro, in vivo,* rodent and human studies, our work represents a comprehensive translational effort to define the discrete biological role of CRFBP. Our results provide converging evidence supporting the role of CRFBP in alcohol consumption.

First, we sought to establish a CRFBP role in CRF-receptor signaling. Here we describe a novel approach where, by tethering human CRFBP with its receptors, we were able to express it at the cell surface and evaluate if CRFBP participates in CRF signaling. We discovered that only CRFR2α is involved in the CRF-mediated receptor activation in the presence of CRFBP. This observation is consistent with our previous electrophysiological work showing that CRF selectively increases *N*-methyl-d-aspartate currents via CRF interaction with CRFR2α and CRFBP.^9^ Furthermore, our results are in line with more recent work demonstrating that CRFBP interacts with CRFR2α and increases receptor expression on the cell surface.^29^ The chimeric receptor approach allowed us to study not only the native protein, but also its fragments. Receptor signal response was detected only when CRFBP(10 kD) was tethered to CRFR2α. By creating a series of chimeric receptors tethered to individual CRFBP fragments, we discovered a potential activating role of the CRFBP(10 kD), specifically via CRFR2α signaling, that would not have been discovered without this approach. The fact that CRFBP(10 kD) tethered to CRFR2α was able to potentiate receptor signaling indicates that CRFBP(10 kD) may play a role *in vivo*. We propose that CRF interacts with CRFBP(10 kD) and CRFR2α in an allosteric manner since CRFBP(27kD) retains the active picomolar affinity binding site for CRF.^30^ As such, our results suggest a potential dual action of CRFR2α and CRFR1 during stress maladaptation, as was previously postulated.^31^ Genetic and pharmacological manipulations targeting CRFR2 have shown that CRF may lead to either increased or decreased response to stressors.^32^ In normal conditions, CRF signal via CRFR1^33^ and CRFBP, in its protecting role, binds the extra unbound CRF.^30^ However, during maladaptive stress induction, CRFBP(27kD) cannot sequester the excess of ‘free' CRF, while CRFBP(10 kD) allosterically potentiates CRFR2α signaling. CRFBP is a critical component of the CRF system, yet its specific physiological role has not been elucidated, mostly because of the difficulty in purifying a large-enough quantity for experimental design.^4^ Our approach is not intended to show that the covalently linked CRFBP-CRFR2α mimics *in vivo* physiological conditions. Rather, this translational approach provides, to the best of our knowledge, the first novel screening assay to identify non-peptidic chemical inhibitors that disrupt the CRFR2α activation in the presence of CRFBP. Our data show an enhancing role of CRFBP and CRFR2α interaction in the central nervous system, a finding consistent with recently reported molecular,^29^ and our original electrophysiology,^9^ data. The coexistence of CRFBP and CRFR2α in glutamatergic/GABA synaptosomes^6^ also supports our findings, as well as independent work suggesting that CRFBP may possess a facilitatory effect on administration of cocaine^8^ and ethanol.^7^ Our chimeric results provide additional evidence to previous work indicating that (1) CRFBP possesses both a sequestering and a potentiating role for CRF;^34^ (2) CRFR2α may play a role in the regulation of stress response;^35^ and (3) ‘free' CRF alone does not necessarily predict stress response.^23^

Previous work showed that CRF is a modulator of ethanol drinking within the DID paradigm.^36, 37, 38^ In the present work, we evaluated the *in vivo* role of CRFBP in binge-like ethanol consumption. For this purpose, we utilized the *CRHBP*−/− mouse integrated in the DID procedure, a validated procedure to study the transition to ethanol dependence. Consistent with the previous characterization of *CRHBP*−*/*− mice,^39, 40, 41, 42^ our results indicated that, in the absence of the CRFBP, higher levels of unbound CRF increase CRFR activation, leading to increased ethanol consumption in the *CRHBP*−/− mice. Our results extend previous findings demonstrating an important role of CRFBP in attenuating the neural responses produced by continuous exposure to stress insults.^30^ These observations are further corroborated, first by a signal detected in the genotype × time interaction. Then, a *post hoc* analysis revealed that *CRHBP*−*/*− mice compared to the *CRHBP+/+* littermates showed increased ethanol consumption after 6-DID exposures. While CRF involvement was not tested directly and is a recognized limitation of this study, we speculate that our results are CRF-mediated as they corroborate previous work using the DID paradigm, where ethanol intake enhances CRF function in the CeA, and systemic, intracerebroventricular and intra-amygdalar administration of CRFR1 antagonist reduces DID intake.^32, 36, 37, 43, 44^ In line with these results, we found here the increase of ethanol drinking behavior observed in *CRHBP*−/− mice after the repeated DID cycle is most likely the result of increased unbound CRF in the brain of *CRHBP*-deficient animals, rather than a basal downregulation of CRF synthesis measured in the CeA and PVN, or an alteration of ethanol metabolism. Our western blot analysis, in ethanol naive mice, are consistent with the recent published data on CRF mRNA levels in *CRHBP*−/− mice and their littermates.^45^ Together, these results support a contribution of CRFBP in ethanol consumption in mice, and raise the possibility that CRFBP imbalance contributes to binge drinking.

CRF mediates behavioral responses to stress and ethanol consumption in extra-hypothalamic regions, including the CeA.^24^ The *in vivo* function of CRFBP in regulating neuronal activity in circuits involved in addiction has been poorly understood due to the instability of CRFBP *in vitro* and the lack of pharmacological probes. Thus, in order to determine the mechanistic role of CRFBP in extrahypothalamic regions and its effect on ethanol-seeking behavior, we hypothesized that downregulation of *CRHBP* in the CeA modulates excitatory behavioral outcomes. This was based on the observation that the CeA is a limbic brain region known to be important for modulation of stress activation where *CRHBP* is highly expressed.^22^ Reduced *CRHBP* levels in the CeA resulted in lower drinking in the ethanol operant self-administration procedure; however, the reduction of ethanol consumption did not persist after a yohimbine challenge. Our results suggest that downregulating CRFBP regional expression, by knocking down CRFBP in the CeA, was sufficient to blunt the ethanol drinking-behavioral phenotype *per se*. However, it is not sufficient to control drinking during activation of the brain norepinephrine system. Consistent with this hypothesis, our fMRI data showed that other known brain regions activated by yohimbine and sensitive to norepinephrine stimulation (caudate putamen and PVN studied here) are activated, and they may be responsible for the increase of ethanol consumption. Viral infusion into the CeA selectively altered the reactivity to yohimbine in this area, but not in other brain structures in *CRHBP* knockdown rats, probably because shRNA *CRHBP* only downregulated CRFBP expression (by 50%), resulting in confined reduction of metabolic activation in the CeA without affecting other brain areas with strong connectivity. These observations are in line with recent results demonstrating that microinjection of CRFBP antagonist CRF_6-33_ into the CeA does not change alcohol intake, while CRFBP in the VTA may be responsible for escalation of ethanol drinking.^7^

Our final goal was to provide initial translational evidence of our findings. While the covalently linked CRFBP-CRFR2α does not represent an *in vivo* physiological condition, our *in vitro* chimeric work guided us in narrowing down the SNP selection from 322 to 88 amino-acid residues. We showed that *rs10062367* A seems to be a risk allele, as it was associated with increased average drinks/day and an increased anxiety trait. These data are not consistent with previous work^26^ where *rs10055255*, but not *rs10062367*, was associated with alcohol- and stress-related phenotypes. However, several factors may explain the differences in these results, for example, differences in the size of the two samples, race- and ethnicity-related differences, and different instruments used to assess the outcomes of the two studies. On the other hand, consistent with the other previous studies,^26, 27^ we found that *rs7718461* A and *rs1053989* C alleles were associated with lower neuroticism and anxiety (reduced risk). However, it is important to note that the SNPs analyzed here are all located in the intronic portion of the gene, which suggests that they might not have functional significance. Although the selected SNPs were all intronic, there are no known SNPs in the gene region encoding for the CRFBP(10 kD) fragment; some are very rare while others have extremely low minor allele to allow for properly powered genetic analyses. Nonetheless, our approach was based on a strong *a priori* hypothesis and our results suggest that CRFBP may play a role in alcohol- and anxiety-related behaviors in alcohol-dependent patients. Although no case−control differences were found and the exact functionality of these SNPs remain uncertain, the present results, together with previous work, suggest a role of CRFBP in alcohol- and anxiety-related phenotypes in a clinically relevant sample of individuals with alcohol dependence. Indeed, the present data represent the largest human study looking at the genetic variant of the CRFBP gene in alcohol dependence.

In summary, CRFBP has been studied for more than two decades, and a variety of hypotheses on its physiological role have been developed. Our findings suggest that CRFBP may possess a dual role: CRFBP(27kD) is responsible for neutralizing CRF effects, while CRFBP(10 kD) has a potential excitatory function. Evaluating the individual functional interaction of each CRFBP fragment with each receptor may elucidate many aspects of stress and AUD, and lead to the development of novel effective treatments.

## Figures and Tables

**Table 1:** Associations between SNPs, alcohol- and anxiety-related phenotypes in subjects with alcohol dependence

*SNP*	*Allele*	N	β	P	*Ancestry*	*Score*	*Risk*
*rs10062367*	A	467	1.310	0.02286	Full	TLFB	Increased
		206	1.616	0.04844	African	TLFB	Increased
		86	4.034	0.04699	European	STAI anxiety	Increased
*rs7718461* a	A	220	−1.457	0.01491	European	CPRS anxiety	Reduced
*rs1053989* b	C	223	−2.050	0.03561	European	Neuroticism	Reduced
		221	−0.298	0.03035	European	CPRS anxiety	Reduced

Abbreviations: CPRS, Comprehensive Psychopathological Rating Scale; SNP, single-nucleotide polymorphism; STAI, State-Trait Anxiety Inventory; TLFB, alcohol time life followback average drinks.

a Proxy for *rs7718461.*

b Proxy for *rs10055255*.
