# Supplementary material for: Defining the role of corticotropin releasing factor binding protein in alcohol consumption
Source: Transl Psychiatry. 2016 Nov 15;6(11):e953–. doi: 10.1038/tp.2016.208 (PMC5314120; doi:10.1038/tp.2016.208)
Supplement: Supplementary Materials and Methods [file tp2016208x1.docx]

**SUPPLEMENTAL INFORMATION***Mice:* Male, mixed genetic background (C57B/6/SV129 mix) mice were generated as previously described and extensively characterized. The *CRHBP* knock-out (-/-) mice were provided to the Ernest Gallo Clinic and Research Center by Dr. Seasholtz. Generation and maintenance of *CRHBP* deficient progeny was achieved by intercrossing homozygous-deficient knockout (-/-) animals and wild-type (+/+) mice, as previously described ^1^. Heterozygous progeny were then crossed with each other to produce progeny that were both homozygous for the disrupted allele and homozygous for the wild type allele. *CRHBP*-/- and *CRHBP*+/+ littermates were used in the behavioral ‘Drinking-in-the-Dark’ (DID) paradigm. Animals were housed either in a reverse 12-hour light-dark cycle (lights off at 1000 hour) or in a normal 12-hour light cycle (light on at 0700 hour). All animals were weaned at P21. Food and water was available *ad libitum*. The room was held at a constant temperature (23 ± 1°C) and humidity (50%).

*Rats:* Male, Long–Evans rats (weighing 200-250 g upon arrival, Harlan Indianapolis, IN) were individually housed in ventilated Plexiglas cages. Rats were housed in a climate-controlled room (21 ± 1°C) on a 12 h light–dark cycle (lights on at 0700 hour). Food and water were available ad libitum, except for short periods during initial training, as outlined below. Following establishment of stable baseline responding, the rats were maintained on a 12-h light/dark cycle and were individually housed after surgical cannulation and lentiviral infusion. Animals were then transferred to the University California, San Francisco (USCF) imaging facility for brain scan.

*Humans:* 1320 individuals (780 patients with current alcohol dependence [AD] and 540 controls) participated in IRB-approved screening and assessment protocols for alcoholic patients and control subjects at the National Institute on Alcohol Abuse and Alcoholism (NIAAA) at the NIH Clinical Center, Bethesda, MD. Exclusion criteria for the control subjects were alcohol dependence and any clinically significant medical illness or major psychiatric disorders such as Bipolar, Major Depressive or psychotic disorders. The exclusion criteria for the AD patients included clinically neuro-psychiatric disorders such as psychosis or dementia. Assessments upon intake included a medical history and physical examination, the Structured Clinical Interview for DSM-IV Disorders (SCID, DSM-IV) ^2^ to diagnose AD and other psychiatric diagnoses, and the alcohol Timeline Follow-back (TLFB) ^3^ to assess current drinking patterns over the previous 90 days before screening. For this analysis, the TLFB was used to calculate the average drinks. Additional assessments used for this analyses included craving measured by the Penn Alcohol Scale (PACS) ^4^, anxiety measured by the Spielberger State-Trait Anxiety Inventory (STAI, trait measure) ^5^ and the Comprehensive Psychopathological Rating Scale (CPRS, state measure) ^6^, severity of dependence measured by the Alcohol Dependence Scale (ADS) ^7^ and severity of withdrawal measured by the Clinical Institute Withdrawal Assessment-Revised (CIWA-Ar) ^7^.

*Materials*

Human CRFBP and human CRF receptors were acquired from Origene (Rockville, MD, USA). Human Embryonic Kidney (HEK293, CRL-1573) cells were acquired form American Type Culture Collection (**Manassas, VA, USA).** Fetal Bovine Serum (FBS), Dulbecco's Modified Eagle Medium (DMEM), and Tris-Glycine gel were acquired from Invitrogen (Carlsbad, CA, USA). AlexaFluor-488, AlexaFluor-594 (IgG_2b_) conjugated antibody, AlexaFluor**-**633 phalloidin, Opti-MEM Reduced Serum, and Lipofectamine 2000 were acquired form Invitrogen (Carlsbad, CA, USA), monoclonal anti-HA.11 (IgG_1_) antibody from Covance (Princeton, NJ, USA), anti-M1 FLAG antibody from Sigma-Aldrich (St. Louis, MO, USA), anti-CRF-BP (B-8, sc-28373, lot #K0804) and anti-CRF-BP (C-19, sc-1822, lot#D2110) from Santa Cruz Biotechnology, Inc. (Santa Cruz, CA, USA); DAPI (4',6-diamidino-2-phenylindole) to label the nuclei was acquired from Vector Laboratories (Burlingame, CA, USA). CRF, and CRF-R2 specific inhibitor, Antisauvagine 30 (AS-30, # A4727) were acquired form Sigma-Aldrich (St. Louis, MO, USA), FLIPR Calcium Assay Kits were purchased from Molecular Devices (Sunnyvale, CA, USA), and 96-well plates (black wall, clear bottom, BIOCOAT, # 08774256) were purchased from Thermo Fisher Scientific (Waltham, MA, USA). 2-hydroxy-ethylpiperazine-*N*-2-ethane sulphonic acid (HEPES), DL-dithiothreitol, tricine, magnesium chloride (MgCl_2_) ethylenediaminetetraacetic acid (EDTA) and saponin were purchased from Sigma-Aldrich^®^ (St. Louis, USA). Complete mini protease inhibitor cocktail tablets were purchased from Roche (Indianapolis, USA). Wheatgerm agglutinin SPA Beads were purchased from Amersham (Little Chalfont, England) Biosciences. Yohimbine (Sigma-Aldrich, St Louis, MO) was dissolved in distilled water and administered at a dose of 2-mg/kg in a volume of .5ml/kg intraperitoneally (i.p.).

*Cell culture and generation of stably transfected fusion plasmids:* The coding sequences of both proteins were amplified by polymerase chain reaction and subcloned into the pcDNA 3.1 vector. All amplified products were completely sequenced. Both CRFBP and CRFRs have N-terminal epitope tags, FLAG-CRFBP and hemagglutinin (HA)-tagged CRFR1 were previously described ^8^. Briefly, the resulting construct was transfected into HEK293 cells. Stably transfected clones were selected by flow analysis cytometry sorting. For generation of stable cell lines, single colonies were chosen and propagated in the presence of 200mg/ml hygromycin. We made the following chimeras from the CRFBP full length: FLAG-CRFBP(FL)-HA-CRFR2 and FLAG-CRFBP(FL)-HA-CRFR1; from the CRFBP C-terminal fragment of 9.6kD: FLAG-CRFBP(10kD)-HA-CRFR2 and FLAG-CRFBP(10kD)-HA-CRFR1 and from the N-terminal fragment of 27kD: FLAG-CRFBP(27kD)-HA-CRFR2 and FLAG-CRFBP(27kD)-HA-CRFR1. The generated cell clones were maintained in DMEM with 10% fetal bovine serum and 200mg/ml hygromycin. For fluorescent calcium assays with adherent cells, cells were plated on poly-D-lysine-coated plates on the previous day at a density of 40,000 cells per well for G*_q_* based assays using 96-well plates.

*Genotyping*

*Mice:* Offspring of heterozygous mating pairs were genotyped by detection of the disrupted allele in genomic DNA prepared from ear biopsies. Multiplex PCR analysis was performed as previously described ^1^.

*Humans:* Genotyping was conducted at the NIAAA Laboratory of Neurogenetics. Genomic DNA was extracted from whole blood using standard protocols. DNA samples were genotyped using the Illumina OmniExpress BeadChip (Illumina Inc, San Diego, CA, USA) including more than 700 000 SNPs. The average genotype reproducibility was 0.99994. Hardy-Weinberg tests for the three selected SNPs, in the full sample, as well as split by European and African Ancestry, indicated that *rs1053989* and *rs7718461* are not in HWE, which might be expected in a population that was not randomly selected (i.e., it was enriched for AD). Additionally, *rs1053989* and *rs7718461* showed quite different allele frequencies in the European ancestry and African ancestry groups, reasons for why we considered ancestry when looking at associations between these SNPs and the phenotype data. For each phenotypic assessment presented here, results were presented for the full sample, as well as split by European and African ancestry based on subject self-report. We further controlled for ancestry, even when splitting the groups, by including ancestry informative maker scores (AIMS) for European and African ancestry. Ancestry informative markers (*n*=2500) were extracted from the Illumina array to calculate ancestral proportions for all study participants. Using methods described previously for an AIMs panel including 186 markers ^9^, which were not available for the current data set, the ancestry assessment identified six ethnic factors (Africa, Europe, Asia, Far East Asia, Oceania, and Americas). Scores for African and European ancestry were included as covariates in all analyses, even when split by self-reported ancestry. Additionally, all analyses were conducted controlling for the Childhood Trauma Questionnaire (CTQ) total score, as childhood trauma has been found to have strong associations with many phenotypes within this sample, as previously described ^10^.

*Drinking-in-the-dark (DID) procedure:* Male, mixed genetic background (C57BL/6/SV129 mix) mice were all weaned at P21 and entered in the DID cycle protocol at greater than 6 weeks of age. Animals were bred in the reverse light/dark cycle and individually housed in double-grommet cages following weaning. For the two weeks prior to ethanol exposure, two bottles of filtered water were available *ad libitum*. After the two week acclimation period for all mice, *CRHBP*-/- and *CRHBP*+/+ littermates were given access to one bottle of 20% (v/v) ethanol for two hours, for three consecutive days; access began three hours into the dark cycle. On the fourth consecutive day, mice were given access to one bottle of 20% (v/v) ethanol for four hours, three hours into the dark cycle. At all other times, two bottles of filtered water was available *ad libitum*. Ethanol was presented in 50ml plastic centrifuge tubes (Fisher Scientific, Pittsburgh, PA) with a fitted rubber stopper and 2.5-inch stainless steel, double ball bearing sipper tubes. Ethanol consumption was calculated by weighing the ethanol bottles prior to presentation, after two hours of ethanol exposure, and after four hours on the fourth day in the series. Measurements were taken to the nearest .01g. The procedure was repeated each week with three to four days between cycles. The ethanol bottle presentation was alternated between sides of the cage each week. The DID assay was repeated for a total of six cycles for one group of animals. This DID procedure was used due to the high intake of ethanol on the fourth day of access, as reported previously ^11^.

*Mice, water and sucrose consumption*: were measured in separate groups of *CRHBP-/-* and *CRHBP+/+* littermates, greater than 6 weeks of age, that had completed two cycles of the DID. These animals were bred in the normal light/dark cycle, weaned at P21, and group-housed before they were transferred to the reverse light/dark cycle. Once animals were transferred to the reverse light/dark room, they were individually housed in a double-grommet cage and given two weeks to adjust to the new light cycle and the individual housing. For water consumption, the DID paradigm was implemented as described above. Following ethanol exposure on the first day of the second week, two bottles of filtered water were weighed and placed onto the cage overnight. Prior to ethanol exposure the following day, the water bottles were removed and weighed again. Thus, water consumption was measured for the remaining twenty-two hours of the day following ethanol exposure on days one, two, and three in the DID paradigm and for the remaining twenty hours of the day following ethanol exposure, on day four of the paradigm. Measurements were taken to the nearest 0.01 g. A similar procedure to the ethanol DID was used to determine sucrose consumption. Briefly, 5% (v/v) sucrose was presented for two hours, three hours into the dark cycle, for the first three days of the DID procedure during week 3. On the fourth day, access to sucrose increased to four hours. Measurements were taken directly prior to exposure and directly after the two-hour and, when applicable, four-hour exposure time points. Measurements were taken to the nearest 0.01g. At all other times filtered water was available *ad libitum*. For determination of blood ethanol content (BEC), a tail blood sample (25μl) was collected immediately after the 4hr ethanol access period and the plasma fraction was siphoned off and stored in a -20°C freezer until the time of assay. Determination of BEC was achieved using an Analox Alcohol Analyzer (Analox Instruments, Lunenburg, MA). For all DID assays, mouse weights were taken daily to determine the adjusted g/kg intake.

*Rats’ self-administration apparatus and training:* Rats’ self-administration testing was conducted in standard operant conditioning chambers (Coulbourn Instruments, Allentown, PA). Details regarding this apparatus have been described elsewhere ([Richards et al, 2008](http://www.nature.com/npp/journal/v37/n4/full/npp2011268a.html#bib75)). Long–Evans rats (*n*=6 per group) were trained to self-administer 20% ethanol without the use of a sucrose-fading procedure, as described previously ([Simms et al, 2010](http://www.nature.com/npp/journal/v37/n4/full/npp2011268a.html#bib86)). All animals were trained to self-administer their respective solution in daily 30-min fixed ratio 3 (FR3) sessions (.1ml per reinforcer) for a minimum of 20 sessions. For ethanol-trained groups, animals not reaching 0.25g/kg intake per session were excluded from further study.

*Lentivirus production:* Lentivirus was produced using the ViraPower Lentiviral Packaging Mix according to the manufacturer’s instructions (Thermo Fisher Scientific). Viral titers were determined by p24 enzyme-linked immunosorbent assay (Zeptometrix, Buffalo, NY, USA) and were approximately 8×10^7^pg/ml for viruses encoding control and *CRHBP* shRNAs.

*Knockdown efficiency:* was determined by infection of HEK293 cells and measuring levels of CRFBP using semiquantitative Western blot and immunohistochemistry 48 h after infection. The blots were probed with anti-CRFBP (B-8) primary antibody (1:100), or GAPD (1:1000) and then the blots were probed with secondary antibody, donkey anti-goat (IgG-HRP) (1:5000) to visualize CRFBP, or goat anti-mouse (IgG H+L-HRP) (1:5000) to visualize GAPDH and detection by enhanced chemiluminescence. Immunoreactive bands were quantified by scanning densitometry (NIH, Image J) ^12^ to determine knockdown efficiency. Immunohistochemistry validation was determined using CRFBP mouse monoclonal antibody against anti-CRFBP (B-8) to label CRFBP and green fluorescence protein (GFP) to visualize GFP-lentivirus. Briefly, HEK293 cells were probed with anti-CRFBP (B-8, 1:15000; Santa Cruz Biotechnologies) antibody to label CRFBP, and mouse monoclonal anti-GFP antibody (3E6, 1:1500; Invitrogen) to label GFP. AlexaFluor-488 conjugated anti-mouse (IgG_2a_) and donkey anti-mouse FITC (1:250, Jackson ImmunoResearch) were used as secondary antibody. The primary antibody was prepared in Blotto solution (5% Donkey Serum, 0.1% Triton X-100, 1mM CaCl_2_, 50mM Tris, pH 7.5, and water) and incubated at room temperature for 1 hour. Cells were washed three times with 50 mM Tris, pH 7.6, 150 mM NaCl, and 1 mM CaCl_2_ (TBSC). The secondary antibody was prepared in Blotto solution and incubated for 1 hour at room temperature. After washing with TBSC four times, the cells were mounted in Vectashield mounting medium. For subsequent behavioral experiments, the modified virus (*CRHBP* shRNA), and control sequence (*Scr* shRNA), were intra-CeA infused in 13 animals (400-450 g at the time of the surgery). After a 10-days recovery from surgery, we tested the drinking behavior of the animals in the ethanol self-administration procedure, as we have previously described (Simms et al., 2010).

*Intra-amygdala infusion of lentivirus:* Rats (400-450g at the time of the surgery) anesthetized with isoflurane were stereotaxically infused with Lenti-*CRHBP* or Lenti-*Scr* bilaterally in the CeA (anteroposterior, - 2.1mm; mediolateral, ± 4.1mm; dorsolateral, −6.08mm). A stainless-steel infuser (30 gauge) connected via polyvinyl chloride tubing to a 10-μl Hamilton GASTIGHT syringe was used to infuse 0.7–1.0 L of virus at a rate of 0.1μl/min for 7 or 10min. After an additional 7 min, the infuser was removed, and the scalp was closed with sutures. GFP-positive cells indicating viral infection were analyzed for the intensity of CRF immunoreactivity. Bilateral infection in the amygdala was confirmed by MRI scan, and post-mortem histological examination of each animal.

*MRI data acquisition:* MR imaging was performed on a 7 Tesla (310mm bore size) superconducting magnet equipped with actively shielded imaging gradients (400mT/m maximum gradient strength, 120mm inner bore size) (Agilent Technologies, Palo Alto, CA, USA). A 63mm inner diameter quadrature ^1^H birdcage resonator was used for RF pulse transmission and 20mm surface coil was used for signal reception. To prepare the rats for the *in vivo* experiments, the animals were anesthetized with 2% isoflurane in oxygen delivered to a nose-mask and then maintained with a mixture of 1.2-1.5% isoflurane in oxygen for the MR scans. The surface coil was placed on the rat head covering the brain area and positioned in the middle of the volume coil. The position was checked with a localizer sequence and localized shimming on the brain was performed with a 3D field-mapping sequence provided by the manufactures’ VnmrJ 3.1 scanner software (Agilent Technologies, Palo Alto, CA, USA). High-resolution T2-weighted images, which served as an anatomical reference images, were acquired using a fat-suppressed multi-slice 2D turbo spin echo (TSE) sequence: TR = 3 s, effective echo time (TE_eff_) = 36.1ms, number of echoes = 8, FOV = 28.8 x 14.4mm^2^, matrix = 256 x 128, resolution = 112.5 x 112.5µm^2^, 6 axial slices (center slice through amygdala), slice thickness = 1mm, number of averages = 8, total acquisition time (t_AQ_) = 6min 30s.

For fMRI, we applied a fat-suppressed T2*-weighted single shot echo planar imaging (EPI) sequence with outer volume saturation (five saturation slices using slice selective adiabatic pulses suppressing the tissue signal outside the brain). Six axial slices through the amygdala area of the rat brain were acquired, co-registered to the T2-weigted TSE dataset. Further parameters: TR = 10s, TE_kzero_ = 10.8ms, FOV = 28.8 x 14.4mm^2^, matrix = 96 x 48, in-plane resolution = 300 x 300µm^2^, slice thickness = 1mm, BW = 350kHz, number of averages = 1. For the fMRI protocol we acquired in total, 540 EPI scans which resulted in a total measurement time of 1.5h (using TR = 10s). The 540 scans consisted from 3 cycles: The first 180 scans (30min) were acquired while no substance was administered (baseline), the following 180 scans (30min) while saline solution was infused intraperitoneal (i.p.) and 180 scans (30min) while yohimbine (2mg/kg) was given i.p to the animal.

*fMRI data post-processing:* The fMRI data was post processed with in-house written software programmed in Matlab (Matlab 2007a, Mathworks, Natick, MA, USA). The first five datasets of each acquisition cycle (Baseline, Saline and Drug) were rejected due to technical reasons (signal changes due to gradient system heat up), leaving 175 scans for each cycle for further processing. The datasets were exported as Nifti-files and image registration was performed with FLIRT from the FSL package using the first fMRI dataset as a reference ^13^. The fMRI datasets were then co-registered and interpolated to the high resolution TSE images (matrix = 256 x 128). A 2D Gauss filter (2.5 pixel size) was applied to smooth the fMRI data. For further analysis, we selected 125 scans per cycle (20.8min) based on the pharmacodynamics of noradrenergic activation by yohimbine, evaluated from its pharmacokinetics profile. In rodents, yohimbine i.p. administration is readily absorbed, widely distributed and after 10min, 54-95% is retained in the CNS ^14^. The ROIs were drawn manually to investigate the fMRI signal change over time. In three 3 different brain regions, 3 successive slices were selected on the co-registered TSE images to analyze the fMRI signal from the left amygdala (ROI1), the right amygdala (ROI2) (each covering an area of 1.8mm^2^) and the paraventricular nucleus hypothalamus (ROI3), (area = 1.9mm^2^). For the caudate putamen (ROI4) (area = 6mm^2^) all six successive slices were selected. The signal from the Amygdala (ROI1 and RO2) was averaged (because of expecting similar BOLD response) and normalized to the first dataset to calculate the relative fMRI signal change in percent. FMRI data processing was carried out using FEAT (FMRI Expert Analysis Tool) Version 5.98, part of FSL (FMRIB's Software Library, www.fmrib.ox.ac.uk/fsl). Z (Gaussianised T/F) statistic images were thresholded using clusters determined by Z>2.3 and a (corrected) cluster significance threshold of *P*=0.05 ^15^.

*Cell selection and flow cytometry:* Cells were grown for selection in 10% FBS in DMEM cell media with hygromycin selection reagent (concentration of ~106 cells/ml). Stably-transfected cells expressing either FLAG- or HA-tagged proteins were stained using 10mg of unlabeled M1 or HA antibody/106 cells as a 2X cocktail in DMEM/FBS. Resuspended cell pellets were visualized using DMEM/10% FBS plus goat anti-mouse (IgG_2b_) AlexaFluor-488 for FLAG and goat anti-mouse (IgG_1_) and AlexaFluor-594 for HA (1:500). Flow cytometry was performed with a Cytopeia InFlux flow cytometer (BD, Franklin Lake, NJ, USA) equipped with a Coherent Sapphire 488 nm and a Coherent 635nm laser (Santa Clara, CA, USA).

*Image analysis:* HEK293 cells expressing CRFBP and chimeric receptors were imaged using a Zeiss LSM 510 META laser confocal microscope (Zeiss MicroImaging, Thornwood, NY, USA) with Plan-Neofluar 63x/1.4 oil objective, and the factory recommended settings and configurations. Stacks of three-channel confocal images with increments of 0.5mm were taken and settings were kept unchanged during this study. Fluorescence detection channels were configured for: the AlexaFluor-488 (Argon 488nm laser); AlexaFluor-594 and AlexaFluor-633 (He-Ne 543nm laser); and DAPI for nuclear staining, MIRA-900F/Verdi V5 Ti-Sapphire (790nm laser, ~350nm, 2ph. Ex) (Coherent, Santa Clara, CA, USA). Images were analyzed using Imaris Neuroscience software pack (v.7.1.1, Andor Technology, Belfast, Northern Ireland).

*Quantification of receptor surface expression by ELISA:* The set of expression-matched chimeric cell lines generated had the receptor number determined in single- and double-expressing cells using an ELISA assay. 50,000 cells expressing either HA-CRFR2 only, or HA-chimeric proteins, were plated in a 96 well plate in complete growth media. The next day, the media was aspirated and 100ml of fixative was added to each well and incubated for 20min. Fixative was then aspirated and washed 3 times with TBSC. 100ml of freshly prepared Blotto solution (5% BSA (IgG free), 100mM CaCl_2_, 1M Tris, pH 7, and water) was added to each well and incubated for 30min. Blotto was aspirated and primary antibody (HA 1:1000, IgG_1_ 1:1000) in Blotto solution was added to each well and incubated for 1hour. The primary antibody was aspirated and washed 3 times with TBSC. Secondary antibody in 50ml of Blotto solution (5% BSA (IgG free), 100mM CaCl_2_, 1M Tris, pH 7.5, 10% Triton X-100, and water) was added to each well and incubated in the dark for 1hour. The wells were washed 3 times with TBSC, and then 20ul of Vectastain was added. To assess only surface-expressed receptors, cells were permeabilized as described previously ^16^. HEK293 cell lines, chimera cell lines without treatment, and treated with only secondary antibodies were utilized as controls for each experiment. Finally, the plate was read using Spectramax microplate reader (Molecular Devices, Sunnyvale, CA). Solutions were prepared fresh and experiments were carried out at room temperature. Exact cell count per well was obtained to normalize the surface receptor expression and RFU was normalized by the number of cell passages (*n*=1-21).

*Fluorescence-based calcium assay:* We also utilized intracellular calcium mobilization of hCRFRs in HEK293 cells as a well-characterized approach to assess activation and inhibition of CRF-induced signaling ^17,18^. HEK293 cells stably expressing the chimeras (CRFBP-CRFR2 or CRFBP-CRFR1), individual receptors (CRFR2 or CRFR1) and the CRFBP were maintained at 37ºC humidified in 7% CO_2_/93% air atmosphere in DMEM/10% FBS cell media with hygromycin (0.4 %) selection reagent. The selected hygromycin-resistant cells were plated in 96-well clear bottom black microplates at a density of approximately 40,000 cells/well in DMEM/10% FBS media and maintained at 37ºC, 7% CO_2_ for 48 hours prior to calcium mobilization assay. On the day of the calcium assay, the cell media in the microplates was replaced with DMEM/1% FBS media (100μl/well) for 2 hours before cells were loaded a membrane permeable calcium sensitive fluorophore dye (FLUO-3; Molecular Devices, Sunnyvale, CA) diluted in assay buffer (HANKS Balanced Salt Solution supplemented with 20mM 4-(2-hydroxyethyl)-1-piperazineethanesulfonic acid (HEPES), 2.5mM probenecid, pH 7.4; 100μl/well) and incubated at 37°C for 60 minutes, according to the manufacturer’s instructions. The calcium mobilization assay was performed using the FlexStation apparatus (Molecular Devices, Sunnyvale, CA) with the following acquisition settings: excitation = 485nm; emission = 525nm; 21^o^C.

Following a pre-assay baseline reading of each well, the intracellular calcium release in HEK293 cells expressing each chimera, individual receptor, or CRFBP only was measured following CRF application. Data were acquired at 1 pt/s for 20s prior to CRF addition and for 120s following CRF addition (1pM-10mM; 50 μl/well of 5X CRF solutions in assay buffer; each concentration in triplicate).

Additional experiments revealed that dye-loaded cells produced stable CRF-dependent signals, with constant signal amplitudes and CRF sensitivity for up to 6 hours after dye loading. For the inhibition experiment in HEK293 cells expressing CRFBP(10kD)-CRFR2α or CRFR2α, the selective CRFR2 antagonist, antisauvagine 30 (AS-30; 10pM-100μM) was added to the wells (2μl of 100X AS-30 solutions in assay buffer added to a total volume of 200ml per well; each concentration in triplicate) and incubated for 30min prior to measurement of intracellular calcium release by CRF (1μM). The assays were performed with a minimum of 20 different stable cell lines expressing CR-BP-CRFR2 chimera proteins and 10 different stable cell lines expressing CRFBP-CRFR1 chimera proteins with replicate measurements (*n* = 3-4) for each experiment.

*Western blot analysis:* The paraventricular nucleus (PVN) of the hypothalamus and central nucleus of the amygdala (CeA) were dissected and immediately snap-frozen in liquid nitrogen until the time of analysis from naïve adult males (P40). Frozen tissues were mechanically homogenized in ice-cold radioimmunoprecipitation assay buffer containing 50mM Tris-HCl, pH 7.4, 5mM EDTA, 120mM NaCl, 1% NP-40, 0.1% deoxycholate, 0.5% sodium dodecyl sulfate (Invitrogen, Carlsbad, CA, USA) and the protease inhibitor mini tablet (Roche, Indianapolis, IN, USA). After homogenization, samples were briefly sonicated and placed on ice for 30 minutes. Protein concentrations were determined using the BCA protein assay kit (Pierce Biotechnology, Rockford, IL, USA). Samples (25μg) were resolved on NuPAGE 4–12% Bis-Tris gels (Invitrogen, Carlsbad, CA, USA), and transferred onto nitrocellulose membranes for analysis. As a positive control, 400pg of synthetic human/rat CRH (American Peptide Company, Sunnyvale, CA) was also loaded onto the gel. Membranes were then incubated in primary antibody against *pro*-CRH (1:10,000 dilution) (Chemicon, Temecula, CA, USA) or glyceraldehyde-3-phosphate dehydrogenase (GAPDH, 1:5000 dilution) (Santa Cruz Biotechnology, Santa Cruz, CA, USA) in 1% milk in PBS-T, for 4 hours at RT. Anti-goat (HRP)-conjugated antibodies were used as secondary antibodies (1:1000 dilution) (Santa CruzBiotechnology, Santa Cruz, CA, USA). Immunoreactivity was detected via an ECL reaction (ECL Western Blotting, Thermo Scientific Pierce) and Kodak film. The film was digitally scanned for relative densitometric quantification using NIH ImageJ software.

For the chimeric experiment, initial experiments yielded six isolated clones that demonstrated consistent and robust measurable expression of the chimera proteins in the transfected human cell lines as determined by Western blot analysis. The expression of the chimera clones in the cell lines was determined using CRF-BP mouse monoclonal antibody against anti-CRFBP (B-8) to label CRFBP(FL) and CRFBP(27kD), and anti-CRF-BP (C-19) to label CRFBP(10kD) (human origin). 50mg cell lysates were loaded per well on a 4-20% Tris-Glycine gel.

1. Karolyi IJ, Burrows HL, Ramesh TM, et al. Altered anxiety and weight gain in corticotropin-releasing hormone-binding protein-deficient mice. *Proc Natl Acad Sci U S A.* 1999;96(20):11595-11600.

2. First MB, Spitzer RL, Gibbon M, Williams JB. *Structured Clinical Interview for DSM-IV Axis I Disorders: Patient Edition (February 1996 Final), SCID-I/P.* Biometrics Research Department, New York State Psychiatric Institute; 1998.

3. Sobell LC, Sobell MB. Timeline follow-back. *Measuring alcohol consumption*: Springer; 1992:41-72.

4. Flannery BA, Volpicelli JR, Pettinati HM. Psychometric properties of the Penn Alcohol Craving Scale. *Alcoholism, clinical and experimental research.* 1999;23(8):1289-1295.

5. Spielberger CD. *State‐Trait anxiety inventory.* Wiley Online Library; 2010.

6. Perris C, Schalling D, Sedvall G, Åsberg M. Comprehensive psychopathological rating scale-CPRS. *Acta psychiat. scand.* 1978.

7. Sullivan JT, Sykora K, Schneiderman J, Naranjo CA, Sellers EM. Assessment of alcohol withdrawal: the revised clinical institute withdrawal assessment for alcohol scale (CIWA-Ar). *Br J Addict.* 1989;84(11):1353-1357.

8. Bartlett SE, Enquist J, Hopf FW, et al. Dopamine responsiveness is regulated by targeted sorting of D2 receptors. *Proc Natl Acad Sci U S A.* 2005;102(32):11521-11526.

9. Hodgkinson CA, Yuan Q, Xu K, et al. Addictions biology: haplotype-based analysis for 130 candidate genes on a single array. *Alcohol and alcoholism.* 2008;43(5):505-515.

10. Schwandt ML, Heilig M, Hommer DW, George DT, Ramchandani VA. Childhood trauma exposure and alcohol dependence severity in adulthood: mediation by emotional abuse severity and neuroticism. *Alcoholism, clinical and experimental research.* 2013;37(6):984-992.

11. Rhodes JS, Best K, Belknap JK, Finn DA, Crabbe JC. Evaluation of a simple model of ethanol drinking to intoxication in C57BL/6J mice. *Physiol Behav.* 2005;84(1):53-63.

12. Schneider CA, Rasband WS, Eliceiri KW. NIH Image to ImageJ: 25 years of image analysis. *Nat Methods.* 2012;9(7):671-675.

13. Jenkinson M, Bannister P, Brady M, Smith S. Improved optimization for the robust and accurate linear registration and motion correction of brain images. *Neuroimage.* 2002;17(2):825-841.

14. Ho AK, Hoffman DB, Gershon S, Loh HH. Distribution and metabolism of tritiated yohimbine in mice. *Arch Int Pharmacodyn Ther.* 1971;194(2):304-315.

15. Worsley K. Statistical analysis of activation images. *Functional MRI: An introduction to methods.* 2001;14:251-270.

16. Waldhoer M, Fong J, Jones RM, et al. A heterodimer-selective agonist shows in vivo relevance of G protein-coupled receptor dimers. *Proc Natl Acad Sci U S A.* 2005;102(25):9050-9055.

17. Dautzenberg FM, Gutknecht E, Van der Linden I, Olivares-Reyes JA, Durrenberger F, Hauger RL. Cell-type specific calcium signaling by corticotropin-releasing factor type 1 (CRF1) and 2a (CRF2(a)) receptors: phospholipase C-mediated responses in human embryonic kidney 293 but not SK-N-MC neuroblastoma cells. *Biochem Pharmacol.* 2004;68(9):1833-1844.

18. Gutknecht E, Van der Linden I, Van Kolen K, Verhoeven KF, Vauquelin G, Dautzenberg FM. Molecular mechanisms of corticotropin-releasing factor receptor-induced calcium signaling. *Mol Pharmacol.* 2009;75(3):648-657.
