# Supplementary figures and images for: Defining the role of corticotropin releasing factor binding protein in alcohol consumption
Source: Transl Psychiatry. 2016 Nov 15;6(11):e953–. doi: 10.1038/tp.2016.208 (PMC5314120; doi:10.1038/tp.2016.208)

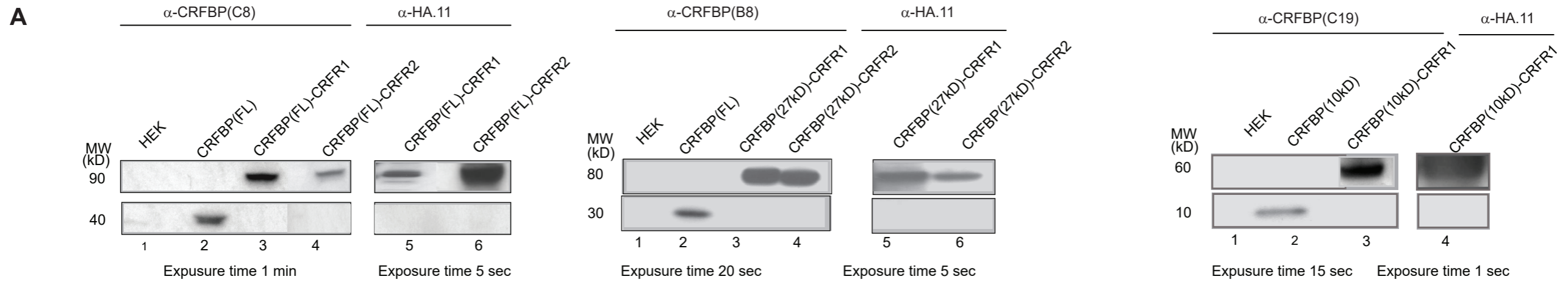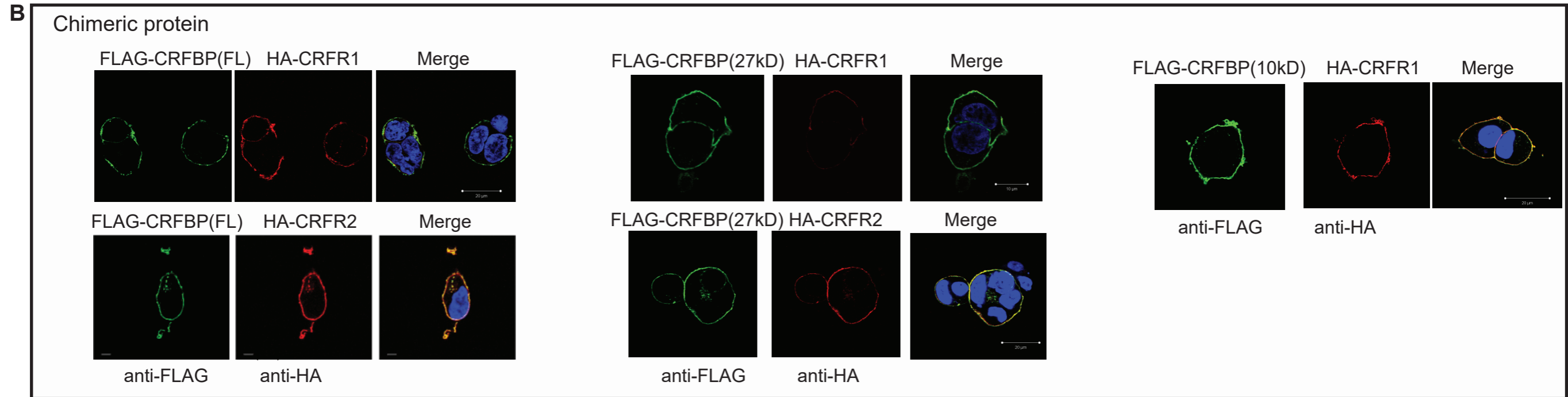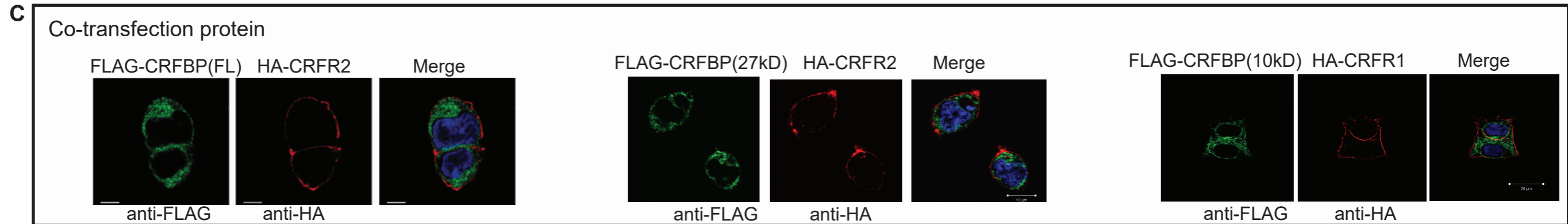

Supplement: Supplementary Figure S1 [file tp2016208x2.pdf]

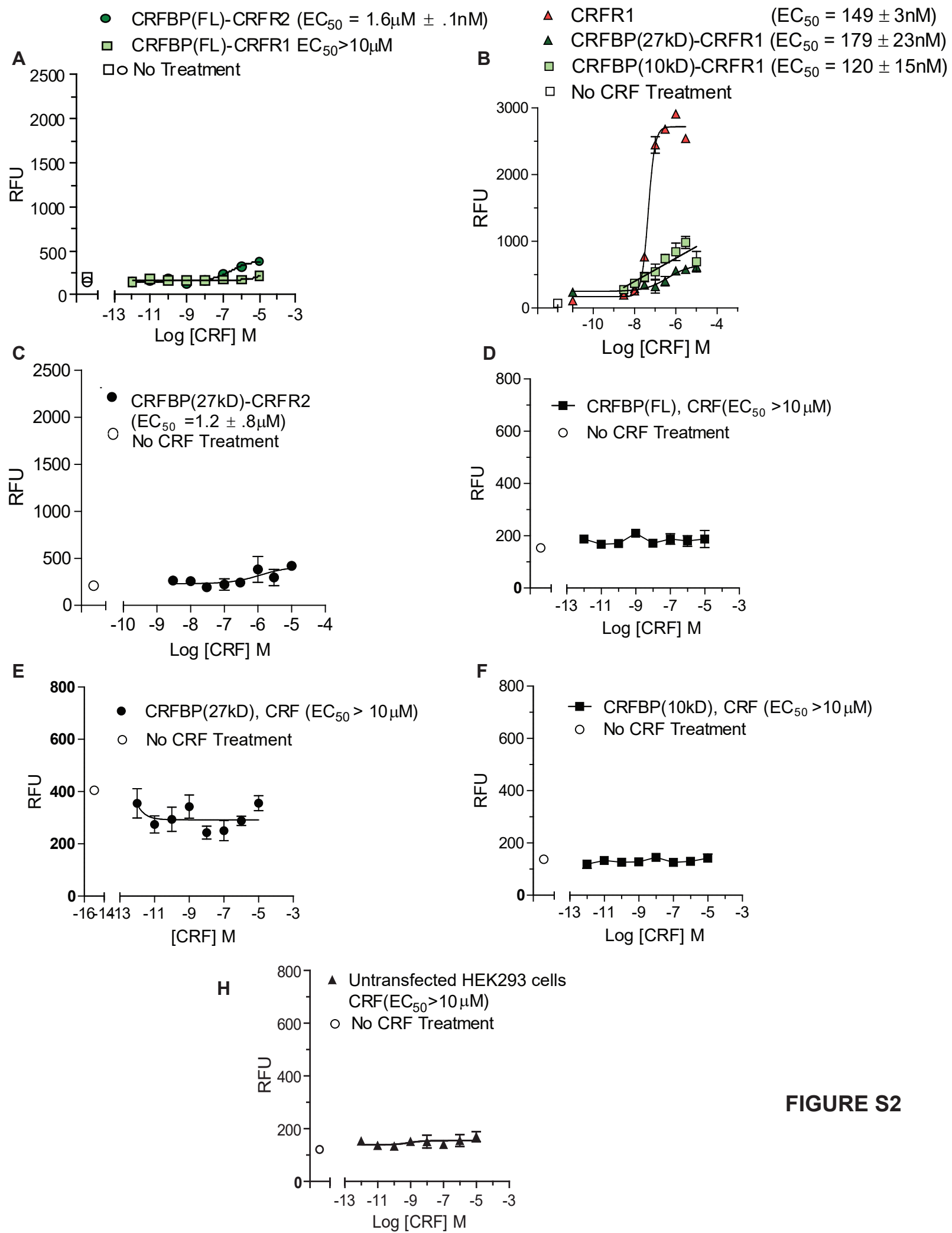

**FIGURE S2**

Supplement: Supplementary Figure S2 [file tp2016208x3.pdf]

# FIGURE S3

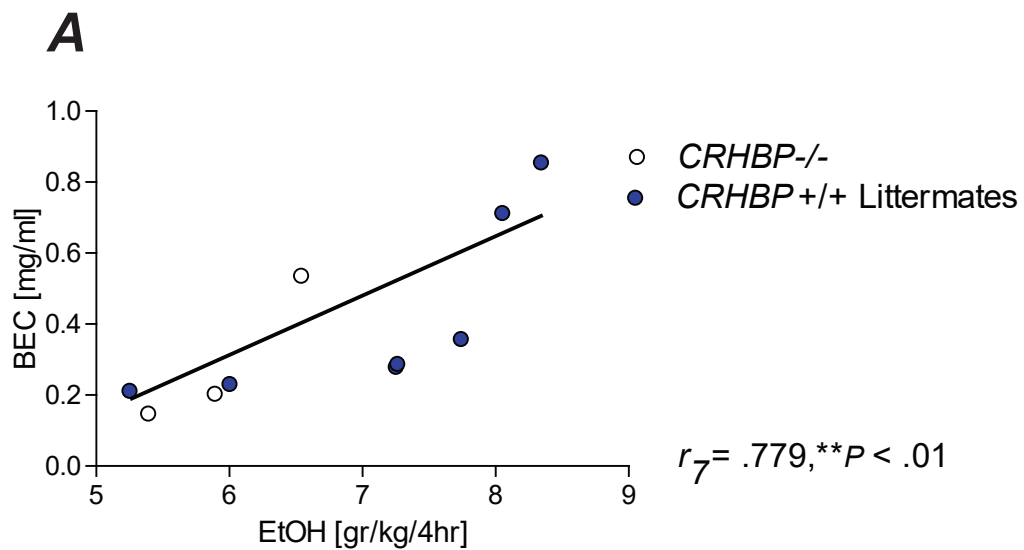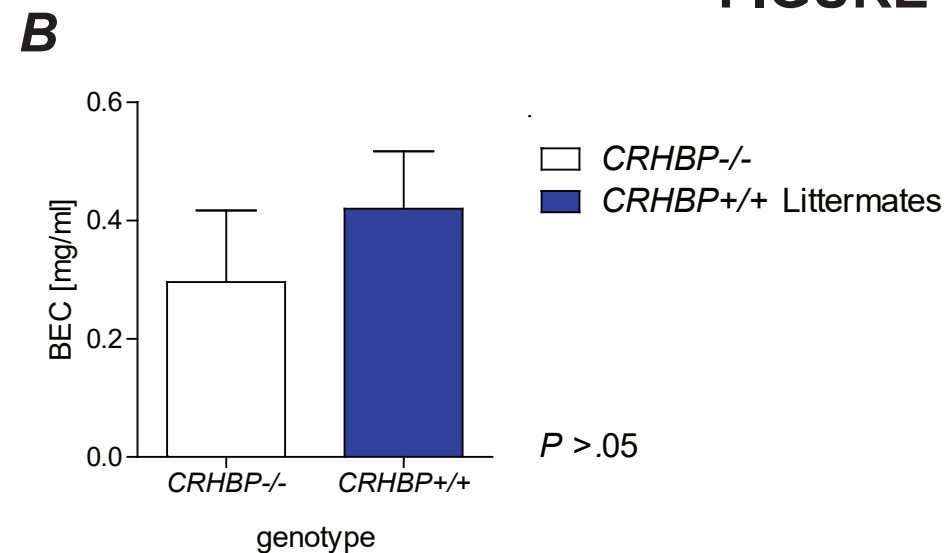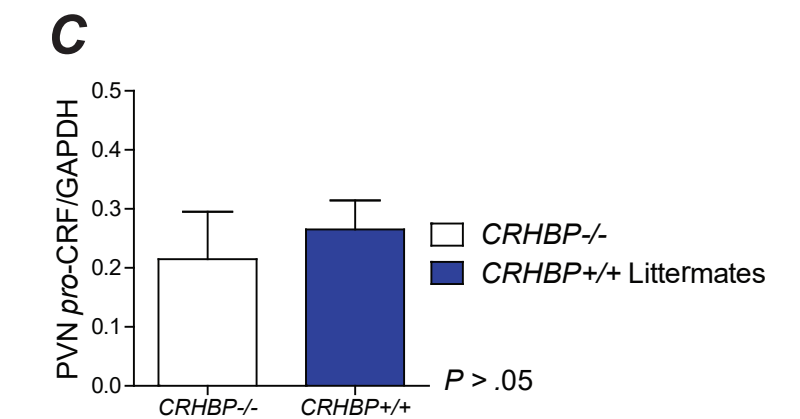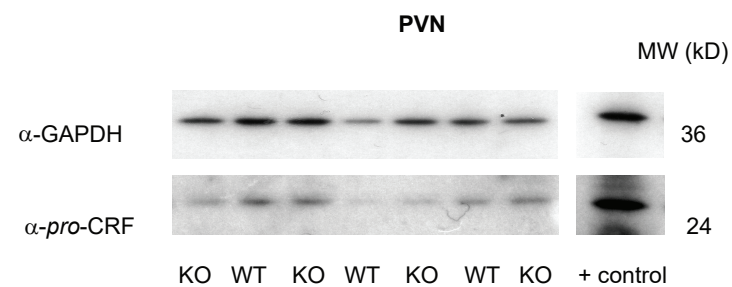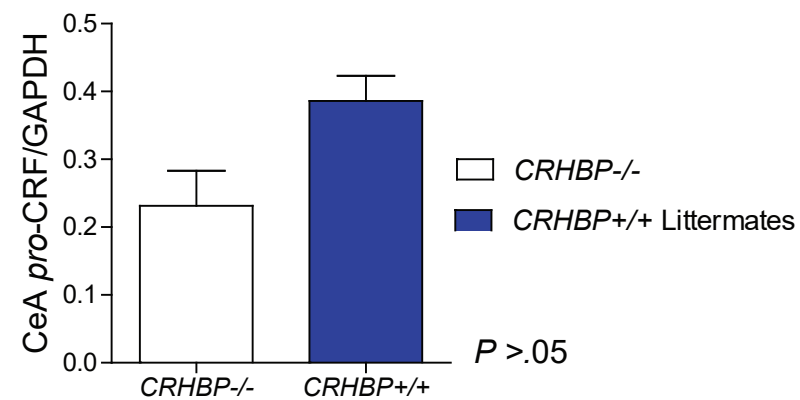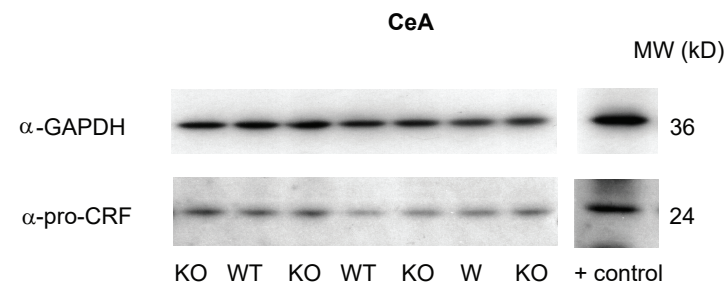

Supplement: Supplementary Figure S3 [file tp2016208x4.pdf]

# FIGURE S4

**A**

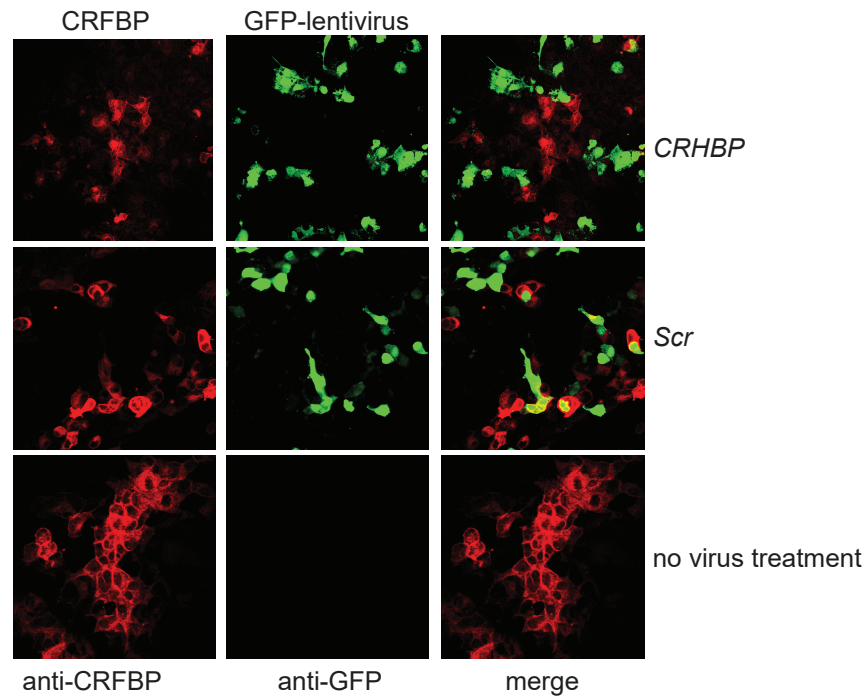

**B**

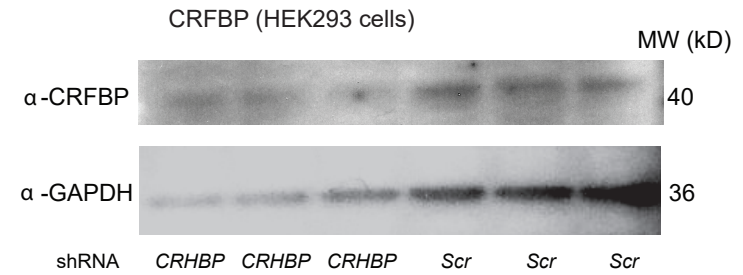

**C**

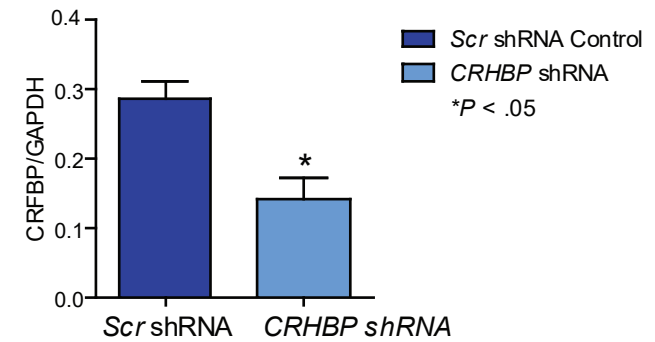

**D**

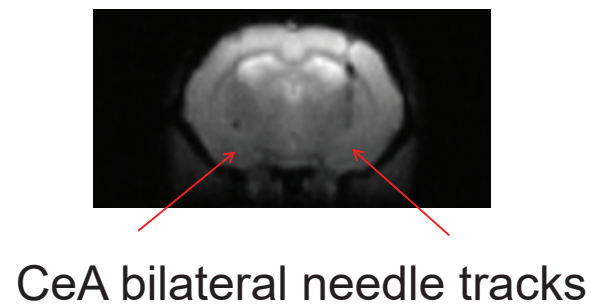

**E**

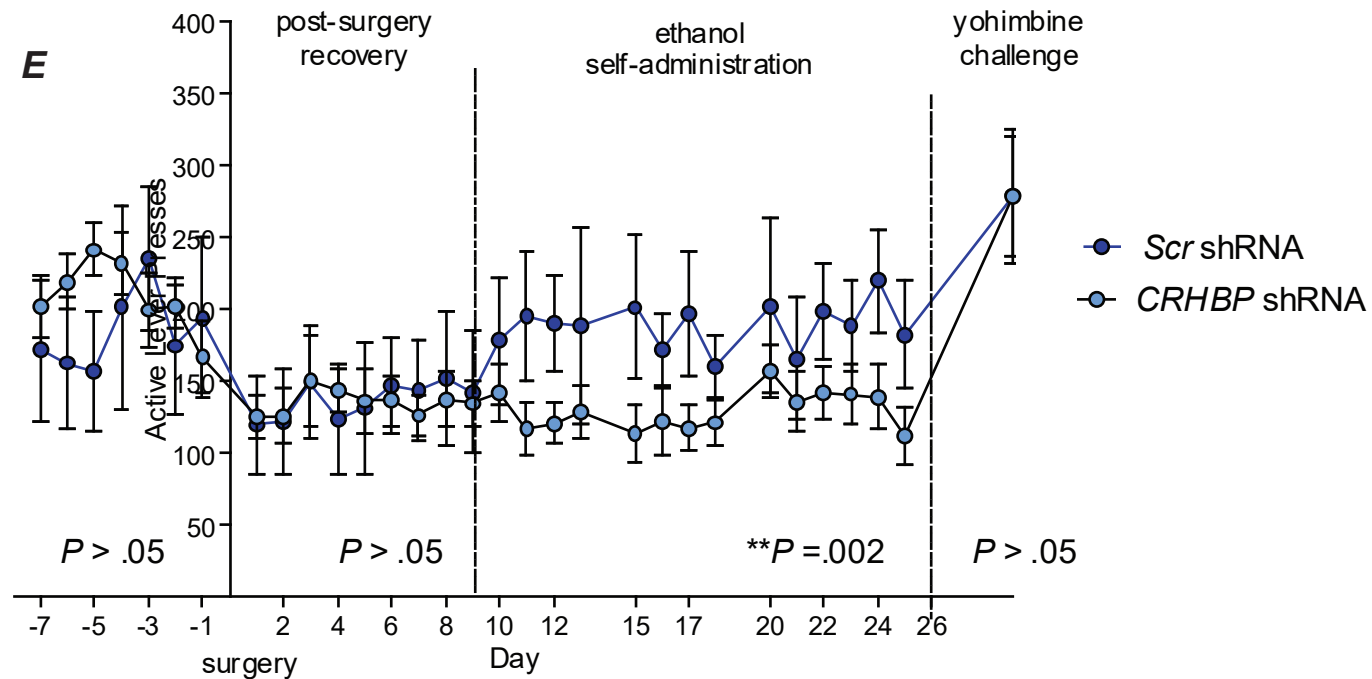

Supplement: Supplementary Figure S4 [file tp2016208x5.pdf]
