# Supplementary material for: Defining the role of corticotropin releasing factor binding protein in alcohol consumption
Source: Transl Psychiatry. 2016 Nov 15;6(11):e953–. doi: 10.1038/tp.2016.208 (PMC5314120; doi:10.1038/tp.2016.208)
Supplement: Supplementary Figure Legends [file tp2016208x6.docx]

**SUPPLEMENTAL FIGURES**

**Figure S1 - The CRFBP(FL)-CRFR1, CRFBP(FL)-CRFR2, CRFBP(27kD)-CRFR1, CRFBP(27kD)-CRFR2 and CRFBP(10kD)-CRFR1 chimeras are expressed on the plasma membrane of HEK293 cells.** (*A*) Western blot analysis of CRFBP from homogenates of HEK293 cells to confirm that chimera proteins are expressed. The blots were probed with anti-CRFBP (C8, B8, C19) primary antibody (1:100), or HA.11 (1:1000) and then the blots were probed with secondary antibody, donkey anti-goat (IgG-HRP) (1:5000), to visualize CRFBP, or goat anti-mouse (IgG H+L-HRP) (1:5000), to visualize CRFRs. FLAG-CRFBP(FL)-HA-CRFRs: Lane 1 (anti-CRFBP): HEK293 cells (negative control), lane 2 (anti-CRFBP): FLAG-CRFBP(FL) (MW~40kD) (positive control), lane 3 (anti-CRFBP): FLAG-CRFBP(FL)-HA-CRF1 (MW~90kD), lane 4 (anti-CRFBP): FLAG-CRFBP(FL)-HA-CRF2 (MW~90kD), lane 5 (anti-HA): FLAG-CRFBP(FL)-HA-CRFR1 (MW~90kD), lane 6 (anti-HA): FLAG-CRFBP(FL)-HA-CRFR2 (MW~90kD). FLAG-CRFBP(27kD)-HA-CRFRs: Lane 1 (anti-CRFBP): HEK293 cells (negative control), lane 2 (anti-CRFBP): FLAG-CRFBP(27kD) (MW~30kD) (positive control), lane 3 (anti-CRFBP): FLAG-CRFBP(27kD)-HA-CRF1 (MW~80kD), lane 4 (anti-CRFBP): FLAG-CRFBP(27kD)-HA-CRF2 (MW~80kD), lane 5 (anti-HA): FLAG-CRFBP(27kD)-HA-CRFR1 (MW~80kD), lane 6 (anti-HA): FLAG-CRFBP(27kD)-HA-CRFR2 (MW~80kD). FLAG-CRFBP(10kD)-HA-CRFR1: Lane 1 (anti-CRFBP): HEK293 cells (negative control), lane 2 (anti-CRFBP): FLAG-CRFBP(10kD) (MW~10kD) (positive control), lane 3 (anti-CRFBP): FLAG-CRFBP(10kD)-HA-CRF1 (MW~60kD), lane 4 (anti-HA): FLAG-CRFBP(10kD)-HA-CRFR1 (MW~60kD). (*B*) Immunohistochemical staining of HEK293 cells transfected with FLAG-CRFBP(FL)-HACRFR1 showing CRFBP(FL) expressed on the cell membrane and co-expressed with CRFR1, FLAG-CRFBP(FL)-HACRFR2 showing CRFBP(FL) expressed on the cell membrane and co-expressed with CRFR2, FLAG-CRFBP(27kD)-HACRFR1 showing CRFBP(27kF) expressed on the cell membrane and co-expressed with CRFR1, FLAG-CRFBP(27kD)-HACRFR1 showing CRFBP(27kD) expressed on the cell membrane and co-expressed with CRFR2, FLAG-CRFBP(10kD)-HACRFR1 showing CRFBP(10kD) expressed on the cell membrane and co-expressed with CRFR1. The cells were fixed and permeabilized and then probed with anti-CRFBP antibodies for FLAG-CRFBP and visualized using AlexaFluor-488 conjugated anti-mouse (IgG_2a_) secondary antibody, or probed using anti-HA and visualized using AlexaFluor-594 conjugated anti-mouse (IgG_1_) antibody and merged fluorescent image with DAPI to visualize the nuclei. (*C*) Immunohistochemical staining of HEK293 cells co-transfected with: CRFBP(FL) and HA-CRFR2, CRFBP(27kD) and HA-CRFR2, CRFBP(10kD) and HA-CRFR1, which do not interact with the receptor at the plasma membrane. FLAG-CRFBP visualized using AlexaFluor-488 conjugated anti-mouse (IgG_2a_) secondary antibody was co-transfected into HEK293 cells with HA-CRFR, probed using anti-HA, and visualized using AlexaFluor-594 conjugated anti-mouse (IgG_1_) and merged fluorescent image with DAPI to visualize the nuclei. Scale bar, 5μm.

**Figure S2. The CRFBP(10kD)-CRFR1, CRFBP(27kD)-CRFR1, CRFBP(27kD)-CRFR2 chimeras and untransfected HEK293 cells are not capable of potentiating CRF-intracellular Ca^2+^ release signaling**. Dose response curves for CRF-induced (1pM-10μM) intracellular calcium release in HEK293 cells expressing the (*A*) FLAG-CRFBP(FL)-HA-CRFR2 chimera proteins produced a CRF dose-dependent increase in intracellular Ca^2+^ release [EC_50_=1.6±01μM] and FLAG-CRFBP(FL)-HA-CRFR1 chimera proteins did not produce Ca^2+^ signaling [EC_50_ > 10μm]. (*B*) FLAG-CRFBP(27kD)-HA-CRFR1 [EC_50_=179±23nM] or HA-CRFR1 [EC_50_=149±3nM] or FLAG-CRFBP(10kD)-HA-CRFR1 [EC_50_=120±15nM], or (*C*) FLAG-CRFBP(27kD)-HA-CRFR2 [EC_50_=1.2±0.8nM], (*D-F*) CRF-mediated calcium signaling was absent in any CRFBP-only transfected, CRFBP(FL), CRFBP(10kD) and CRFBP(27kD), cells and in (*G*) untransfected HEK293 cells. Results are expressed as the *M*±*SEM* RFU, calculated as agonist-induced maximum calcium peak/cell number x 1000.

**Figure S3 - BEC and Western blot analysis of pro-CRF and GAPDH protein expression in the PVN and the CeA**. At week 1, (*A*) there was a linear correlation between ethanol intake and BEC [*n*=8-3, *r*=.779, ***P*<.01] and (*B*) there was no difference in BEC between *CRHBP*-/- and *CRHBP*+/+ littermates [*n*=8-3, *P*>.05]. (*C*) At baseline (week 1 in the DID), in the PVN and CeA, there was no significant differences in *pro*-CRF levels in naïve *CRHBP*-/- mice compared to their *CRHBP*+/+ littermates [*n*=4-3, *P*>.05]. Semiquantitative analysis of *pro*-CRF levels for the PVN and CeA immunoblots, averaged across animals in each group, showed no statistical difference between the two genotypes. *pro*-CRF levels are expressed relative to GAPDH to normalize for protein-loading. Results are reported as *M*±*SEM*; not significant [*P*>.05].

**Figure S4 - Verification of CRFBP downregulation expression *in vitro* and *in vivo*.** (*A*) Immunohistochemistry validation using HEK293 cells transfected with CRFBP and infected with lentivirus expressing GFP and *CRHBP* shRNAs showed a significant reduction of the *CRHBP* expression in the *CRHBP* shRNA group. (*B*) Homogenates from HEK293 cells transfected with CRFBP and infected with either *CRHBP* or *Scr* shRNA lentivirus were used for Western blot analysis. Lane 1: CRFBP (MW~40kD), lane 2: GAPDH (MW~36kD) and (*C*) densiometric quantification of CRFBP protein showed a 50% reduction of the CRFBP expression in the *CRHBP* shRNA group [*t_4_*=3.663, **P*<0.05]. (*D*) Representative of the anatomical MRI scan showing the bilateral needle track in the rat brain to the central nucleus of the amygdala (CeA). (*E*) Before the surgery, during the first 8-sessions following completion of the ethanol self-administration training procedure (baseline ethanol consumption), there was no significant difference for self-administration of 20% ethanol between the groups [*n*=7-6, *P*>.05]. After recovering from surgery, during the subsequent 15 ethanol self-administration sessions, there was a significant shRNA x time interaction [*n*=7-6, *B_1_*=3.546, ***P*<.01]. A subsequent paired *t*-test indicated that rats infected with *CRHBP* shRNA showed a significant reduction in ethanol consumption [*n*=7-6, *t*_69_=8.436, ****P*<.001]. By contrast, in the *Scr* shRNA control rats, there was no statistical difference between the amount of ethanol consumed at baseline and the following sessions after recovering from surgery [*P*>.05]. Results are reported as *M*±*SEM*; not significant [*P*>.05].

**Movie S1. The CRFBP(10kD) is expressed on the plasma membrane and colocalizes with CRFR2 in HEK293 cells.** Immunohistochemical staining of HEK293 cells transfected with FLAG-CRFBP(10kD)-HACRFR2. The cells were fixed and permeabilized, then probed with anti-CRFBP antibodies for FLAG-CRFBP(10kD) and visualized using AlexaFluor-488 conjugated anti-mouse (IgG_2a_) secondary antibody (green), or probed using anti-HA and visualized using AlexaFluor-594 conjugated anti-mouse (IgG_1_) antibody (red) and merged fluorescent image (yellow) with DAPI to visualize the nuclei (blue). Scale bar, 5 μm.
