# Supplementary material for: Defining the role of corticotropin releasing factor binding protein in alcohol consumption
Source: Transl Psychiatry. 2016 Nov 15;6(11):e953–. doi: 10.1038/tp.2016.208 (PMC5314120; doi:10.1038/tp.2016.208)
Supplement: Supplementary Tables [file tp2016208x7.docx]

**SUPPLEMENTAL TABLES**

| **Supplemental Table S1.**  **Ethanol consumption during the repeated DID exposures in the *CRHBP-/-* mice compared to their *CRHBP+/+* littermates** | | | |
| --- | --- | --- | --- |
| *2-hr period* | | | |
| Cycle | *CRHBP*-/- (gr/kg/2hr) | *CRHBP*+/+ (gr/kg/2hr) | *P* |
| 1 | 3.550 ± .219 | 2.778 ± .542 | *ns* |
| 2 | 3.608 ± .271 | 2.312 ± .404 | .004 |
| 3 | 3.502 ± .201 | 2.685 ± .470 | .082 |
| 4 | 4.584 ± .547 | 3.180 ± .112 | .004 |
| 5 | 3.831 ± .339 | 5.482 ± .385 | .010 |
| 6 | 3.747 ± .247 | 2.342 ± .342 | <.0001 |
| *4-hr period* | | | |
| Cycle | *CRHBP*-/- (gr/kg/4hr) | *CRHBP*+/+ (gr/kg/4hr) | *P* |
| 1 | 6.661 ± .454 | 5.853 ± .452 | *ns* |
| 2 | 6.647 ± .262 | 6.619 ± .575 | *ns* |
| 3 | 7.625 ± .358 | 5.775 ± 1.000 | .059 |
| 4 | 7.979 ± .476 | 5.790 ± .638 | .003 |
| 5 | 7.121 ± .573 | 5.482 ± .385 | .010 |
| 6 | 7.750 ± .426 | 5.223 ± .440 | <.0001 |

**Supplemental Table S2.**

**Proxy SNPs in high linkage disequilibrium (LD) with original SNPs**

| **SNP** | **Proxy** | ***R^2^*** | ***D`*** | **Ancestry** |
| --- | --- | --- | --- | --- |
| *rs10055255* | *rs1053989* | .966 | .966 | European |
| *rs10055255* | *rs1053989* | 1 | 1 | African |
| *rs7728378* | *rs7718461* | 1 | 1 | European |
| *rs7728378* | *rs7718461* | .816 | .816 | African |

| **Supplemental Table S3.**  **Allele frequencies of the SNPs selected from the clinical dataset** | | | | | | | | | |
| --- | --- | --- | --- | --- | --- | --- | --- | --- | --- |
|  | | | | Full Sample | | | Full Sample, AD only | | |
| CHR | SNP | A1 | A2 | MAF | NCHROBS | *N* | MAF | NCHROBS | *N* |
| 5 | *rs1053989* | C | A | .4939 | 2636 | 1318 | .4807 | 1558 | 779 |
| 5 | *rs10062367* | A | G | .2468 | 2626 | 1313 | .2500 | 1552 | 776 |
| 5 | *rs7718461* | A | G | .4902 | 2640 | 1320 | .4763 | 1560 | 780 |
|  |  |  |  | European only | | | European, AD only | | |
| CHR | *SNP* | A1 | A2 | MAF | NCHROBS | *N* | MAF | NCHROBS | *N* |
| 5 | *rs1053989* | A | C | .3868 | 1484 | 742 | .3875 | 818 | 409 |
| 5 | *rs10062367* | A | G | .1856 | 1476 | 738 | .1847 | 812 | 406 |
| 5 | *rs7718461* | G | A | .3969 | 1484 | 742 | .4020 | 816 | 408 |
|  |  |  |  | African only | | | African, AD only | | |
| CHR | *SNP* | A1 | A2 | MAF | NCHROBS | *N* | MAF | NCHROBS | *N* |
| 5 | rs1053989 | C | A | .2852 | 894 | 447 | .2929 | 618 | 309 |
| 5 | *rs10062367* | A | G | .3610 | 892 | 446 | .3414 | 618 | 309 |
| 5 | *rs7718461* | A | G | .2951 | 898 | 449 | .3039 | 622 | 311 |

A: allele; AD: alcohol-dependence; CHR: chromosome; MAF: Minor allele frequency; NCHROBS: non-missing allele count
